# Critical functions and key interactions mediated by the RNase E scaffolding domain in *Pseudomonas aeruginosa*

**DOI:** 10.1371/journal.pgen.1011618

**Published:** 2025-03-17

**Authors:** Sandra Amandine Marie Geslain, Stéphane Hausmann, Johan Geiser, George Edward Allen, Diego Gonzalez, Martina Valentini

**Affiliations:** 1 Department of Microbiology and Molecular Medicine, University of Geneva, Geneva, Switzerland; 2 Laboratory of Microbiology, University of Neuchâtel, Neuchâtel, Switzerland; Indiana University Bloomington, UNITED STATES OF AMERICA

## Abstract

The RNA degradosome is a bacterial multi-protein complex mediating mRNA processing and degradation. In Pseudomonadota, this complex assembles on the C-terminal domain (CTD) of RNase E through short linear motifs (SLiMs) that determine its composition and functionality. In the human pathogen *Pseudomonas aeruginosa*, the RNase E CTD exhibits limited similarity to that of model organisms, impeding our understanding of RNA metabolic processes in this bacterium. Our study systematically maps the interactions mediated by the *P. aeruginosa* RNase E CTD and highlights its critical role in transcript regulation and cellular functions. We identified the SLiMs crucial for membrane attachment, RNA binding and complex clustering, as well as for direct binding to the core components PNPase and RhlB. Transcriptome analyses of RNase E CTD mutants revealed altered expression of genes involved in quorum sensing, type III secretion, and amino acid metabolism. Additionally, we show that the mutants are impaired in cold adaptation, pH response, and virulence in an infection model. Overall, this work establishes the essential role of the RNA degradosome in driving bacterial adaptability and pathogenicity.

## Author summary

Bacteria must rapidly adapt to changing environments, whether facing temperature shifts, nutrient scarcity, or antibiotic exposure. A key mechanism enabling this adaptability is the regulation of the cellular levels of mRNAs—the molecular blueprints for protein production. These levels are governed by an equilibrium between RNA synthesis and degradation, the latter action performed by a multi-protein complex called the RNA degradosome. Although the RNA degradosome core function is conserved across bacteria, its composition and organization differ significantly between species, reflecting diverse lifestyles adopted and environmental challenges encountered by each bacterium.

In this study, we investigated the RNA degradosome in *Pseudomonas aeruginosa*, a bacterial pathogen causing difficult-to-treat infections in humans. We identified its components and mapped regions within the complex essential for RNA binding, membrane attachment, and spatial organization. Disrupting these regions compromised *P. aeruginosa* ability to survive in cold conditions, respond to stress, and establish an infection. Through this work, we uncovered unique features of the *P. aeruginosa* RNA degradosome that distinguish it from those of other species, emphasizing the RNA degradosome critical role in bacterial adaptability and highlighting it as a promising target for therapies against *P. aeruginosa* infections.

## Introduction

In bacteria, the processing and degradation of mRNAs is primarily orchestrated by the RNA degradosome, a multi-enzyme complex that includes ribonucleases, RNA helicases, and various other proteins [1–4]. The RNA degradosome plays a crucial role in enabling bacteria to rapidly adapt to changing environmental conditions by swiftly modifying mRNA levels and broadly regulating gene expression [5,6].

Most of our knowledge on the RNA degradosome derives from studies in *Escherichia coli*, where the RNase E endonuclease serves as the central component [7–10]. While the catalytic activity of *E. coli* RNase E resides in its N-terminal domain (NTD, amino acids 1-529), the C-terminal domain (CTD, amino acids 530-1061), or scaffolding domain, is intrinsically disordered and contains short linear motifs (SLiMs) of 10 to 40 amino acids to which the other RNA degradosome components bind [10,11]. Core *E. coli* RNA degradosome components, namely the polyribonucleotide phosphorylase PNPase [12], the RNA helicase RhlB [13], and the glycolytic enzyme enolase [14], interact with RNase E in semi-stoichiometric ratios via the PBS (PNPase binding site), HBS (helicase binding site) and EBS (enolase binding cite) SLiM, respectively [15,16]. The *E. coli* RNase E CTD also includes an amphipathic helix, called membrane targeting sequence (MTS), which tethers the entire complex to the inner membrane, and at least two RNA-binding SLiMs, AR1 (named also RBD) and AR2, which mediate contact with translating ribosomes, small non-coding RNAs and mRNAs [11,17–20]. Additional proteins, such as RraA [21,22], RraB [23], CsdA [24], Ppk [25], MinD [26,27], and Hfq [28], can interact with *E. coli* RNase E in sub-stochiometric ratios and/or under non-standard conditions of growth (reviewed in [29]). Of importance, the RNase E NTD is essential for *E. coli* growth and rRNA processing [30], whereas deletion of the CTD is not lethal and alters mRNA levels without affecting rRNAs [18,31–33]. This highlights the dual role of RNase E, with the NTD being crucial for overall cell maintenance, and the CTD regulating specific RNA substrates through the assembly of the RNA degradosome.

RNase E homologs are present in approximately 49% of sequenced bacterial genomes, and nearly all Pseudomonadota (formerly called Proteobacteria) encode at least one RNase E homolog, with the notable exception of most *Campylobacter* and *Helicobacter* species [4,29]. The RNase E NTD is remarkably conserved across most species, while the CTD varies considerably in sequence, length, and SLiMs composition, even between species belonging to the same order [29]. SLiMs variability is thought to drive the diversity of RNase E interacting partners, and therefore RNA degradosome composition, among bacterial species. For instance, in *Caulobacter crescentus,* RNase E interacts with PNPase, RhlB, RNase D, and the Krebs cycle enzyme aconitase [34,35]. In *Anabaena* sp. strain PCC 7120, RNase E partners with PNPase, enolase, the RNA helicase CrhB, and RNase II [36–38], while in *Pseudomonas syringae* LZ4W, only RNase R and the RNA helicase RhlE were found to co-purify with RNase E in pull-down assays [39]. Similarly, in *Rhodobacter capsulatus*, purification of the RNA degradosome complex via glycerol-gradient centrifugation of cell extracts revealed the presence of RNase E, two unnamed RNA helicases, the Rho transcription termination factor, and two unidentified proteins of 47 and 36 kDa, with only low amounts of PNPase present in the purified fractions [40].

In these examples, the SLiMs mediating protein-protein interactions were not always characterized. Moreover, even when the interaction of RNase E with a certain protein partner is conserved across several species, such as the RNase E-PNPase and RNase E-RhlB interactions, the SLiMs involved in the binding show little sequence identity [29,35,36,41–43]. Therefore, predicting RNA degradosome composition based solely on the RNase E CTD sequence does not seem possible. Experimental characterization of the SLIMs across different bacterial species and their impact on the RNA degradosome composition and function is essential, providing insights into novel regulatory mechanisms that enable bacteria to thrive under stress and adapt to diverse environments.

Little is known about the RNA degradosome composition of the opportunistic human pathogen *Pseudomonas aeruginosa*, which is a leading cause of nosocomial infections worldwide largely due to its remarkable adaptability to different environments and resistance to antibiotics [44–52]. A recent study has revealed that *P. aeruginosa* RNase E co-elutes with PNPase, the PA0428 RNA helicase, and several other proteins in pull-down assays, although their interaction with RNase E has neither been confirmed nor mapped [53]. Our laboratory has characterized the PA0428 RNA helicase, named RhlE2, confirming an RNA-dependent interaction with RNase E and identifying the RhlE2 C-terminal region as necessary and sufficient for this binding [54,55]. However, the specific RNase E interaction site remained unmapped.

In this study, we characterize the SLiMs within the CTD of *P. aeruginosa* RNase E, identifying those involved in RNA binding (AR1, AR4, and REER-repeats) as well as those mediating interactions with core RNA degradosome components, which we determine to be PNPase and the RNA helicase RhlB (NDPR and AR1, respectively). We also show that RhlE2 interacts with the RNase E NTD in pull-down assays. Protein-protein interactions were probed with multiple experimental approaches, including an analysis of the subcellular localization dynamics of the complex. Additionally, we performed transcriptomic and phenotypic analyses of RNase E CTD mutant strains, revealing the critical role of RNA degradosome complex scaffolding in bacterial virulence and adaptation to fluctuations in temperature or pH.

The ability of *P. aeruginosa* to cause disease is intrinsically linked to a sophisticated gene regulatory network that facilitates bacterial survival and virulence. Within this network, the role of post-transcriptional gene regulation remains only partially explored [56,57]. By uncovering the composition and functional dynamics of the RNA degradosome in *P. aeruginosa*, our study not only advances the understanding of the molecular mechanisms driving bacterial environmental adaptation but also reveals potential novel drug targets, which could be crucial in the fight against antibiotic-resistant infections.

## Results

### The P*. aeruginosa* RNase E harbours several uncharacterised SLiMs conserved within the Pseudomonadales

**Fig 1:**
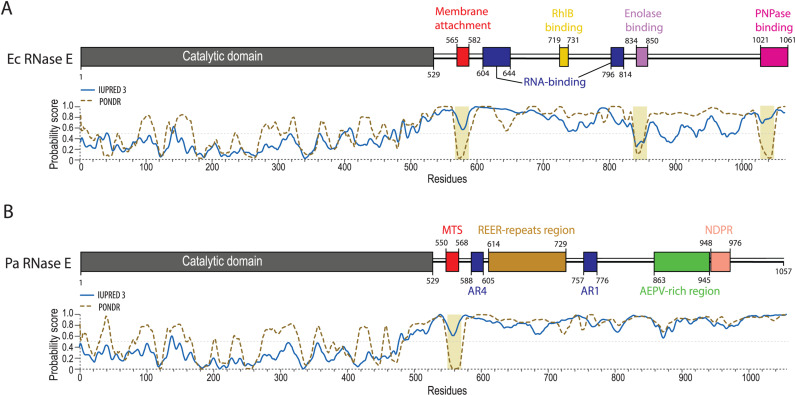
Comparison of *E. coli* (Ec) and *P. aeruginosa* (Pa) RNase E architecture. **(A)** Ec RNase E architecture, adapted from [16]. The NTD is shown is grey, and experimentally defined regions necessary for interaction with the membrane, with RNA, or with the three core protein partners are highlighted in red, dark blue, and yellow/purple/pink, respectively. **(B)** Pa RNase E architecture based on in-silico analyses performed in S3 Fig and in [29]. The SLiM nomenclature was taken from [29], while the consensus sequence characterising each SLiM was specifically adapted based on sequence conservation within the Pseudomonas genus (S3 Fig). MTS: membrane-targeting sequence, AR4: arginine-rich motif 4, REER-repeats: region with high frequency of occurrence of “RE [E/D]R” motif, AR1: arginine-rich motif 1, AEPV-rich region: region rich in AEPV residues, NDPR: motif with a highly conserved NDPR sequence. All diagrams were drawn to scale. IUPRED3 [58] and PONDR [59] predictions of disorder propensity (probability score) are shown for Ec RNase E **(A)** and Pa RNase E **(B)**.

*P. aeruginosa* RNase E (Pa RNase E) consists of an NTD (amino acids 1-529) that shares high sequence identity (70%) and similarity (90%) with the NTD of *E. coli* RNase E (Ec RNase E), and a CTD (amino acids 530-1057) that shows only 25% identity and 37% similarity with the CTD of Ec RNase E (S1 Fig). Previous work by Aït-Bara et al. (2015) identified seven different putative SLiMs on the Pa RNase E CTD (MTS, AR4, REE, M29, AR1, M20 and NDPR) [29], which are shown in comparison to the Ec RNase E in S2A Fig. Of note, the MTS is the only SLiM of Pa RNase E CTD with a propensity to form a secondary structure while in Ec RNase E, the enolase and PNPase binding sites also exhibit this feature (Fig 1 and [11]).

To refine the prediction of Pa RNase E SLiMs, we performed a multiple sequence alignment of *Pseudomonas* RNase E homologs, identifying conserved motifs (S3 Fig). Several highly conserved CTD regions overlap with the SLiMs previously identified, with the notable presence of additional conserved residues located immediately adjacent to these SLiMs (S2A and S3 Figs). Based on these data, we updated the SLiMs boundaries to include these conserved residues (Fig 1B, former SLiMs boundaries in S2A Fig). Upon manual inspection of the CTD sequence, we also observed a large duplicated sequence (residues 635-667 and 682-714) within the CTD region containing the REE and motif 29 SLiMs. Since motif 29 is defined by the conserved motif REERQPR, we merged the two SLiMs and renamed the entire segment (residues 614-729) as the “REER-repeats” region (Fig 1B). The number of REER repeats varies across *Pseudomonas* species: *P. aeruginosa* strains typically harbour 13 RE [ED]R repeats, whereas the *P. fluorescens*, *P. protegens*, *P. stutzeri*, and *P. syringae* environmental species show a distribution of repeats peaking around 8-10. The exception is *P. putida*, which exhibits a high frequency of 20-23 RE [ED]R repeats (S2B Fig). This variability suggests a high frequency of indels occurring within the REER-repeats region.

Overall, our in-silico analyses of Pa RNase E CTD sequence refined the number and length of the Pa RNase E SLiMs, highlighting a potential functional significance of the REER-repeats region in *Pseudomonas* species, and suggesting differences in RNA degradosome complex dynamics between *P. aeruginosa* and *E. coli*.

### The REER-repeats, AR1 and AR4 SLiMs mediate RNase E CTD RNA binding and foci formation

**Fig 2:**
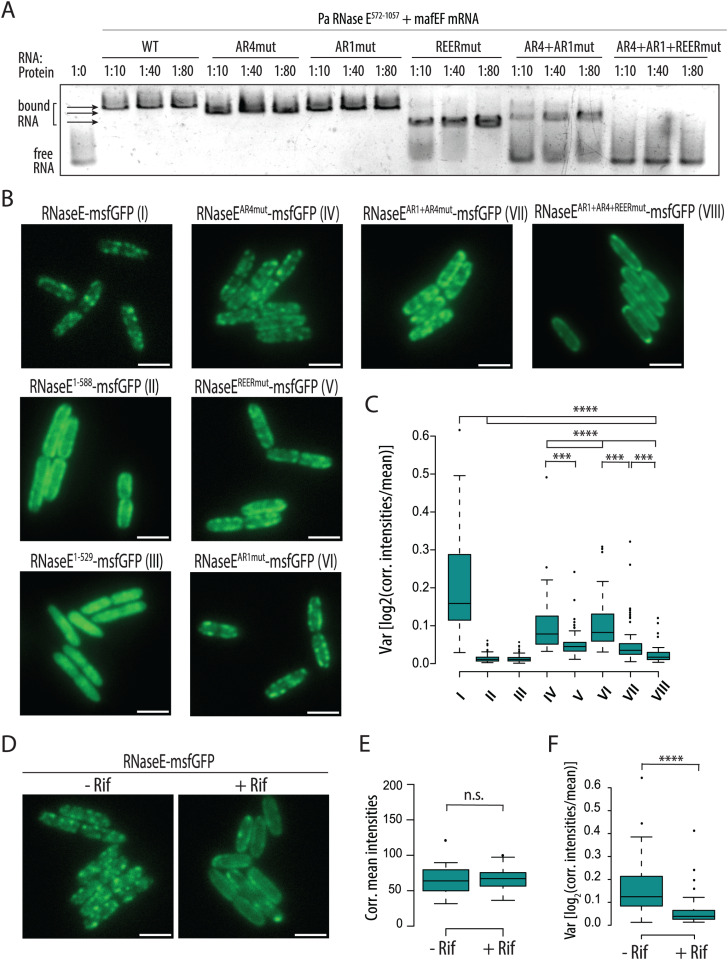
Pa RNase E CTD binds RNA via the AR1, AR4, and REER SLiMs. (A) EMSA with purified His_10_-Smt3 FLAG-tagged RNase E CTD^572-1057^ variants having either the native sequence (WT) or systematic alanine mutation of the arginine residues found within the REER-repeats region (REERmut), the AR4 SLiM (AR4mut) or the AR1 SLiM (AR1mut). The *malEF* mRNA intergenic region was used as a substrate [61] at a final concentration of 35 nM (see Materials and methods). Molar ratios of RN A:protein ranging from 1:10 to 1:80 were tested. See S4A and S4B Fig for additional EMSA with additional RNA:protein ratios. **(B)** Representative microscopy images of a PAO1 strain expressing the RNase E-msfGFP fusion or mutated or truncated variants as indicated. **(C)** Quantification of normalised signal intensity variance in the strains shown in panel B as indication of foci disappearance (see Materials and methods). **(D)** Representative microscopy images of a PAO1 strain expressing the RNase E-msfGFP fusion after a 30-minute exposure to rifampicin (+ Rif, exposure to a concentration of 100 μg/mL) or DMSO as a negative control (- Rif). Corrected mean fluorescent signal intensity **(E)** and signal intensity variance **(F)** are shown in the graphs (see Materials and methods). Each quantification analysis involved at least 50 individual cells. **(B-D)** The scale is 2 μm. Statistical significance was assessed by performing t-tests using R. p-values for comparisons tested are indicated as follows: *:<0.05; **:<0.01; ***:<0.001; ****:<0.0001.

Previous works in *E. coli* identified arginine-rich SLiMs as RNA-binding motifs [20,60]. We therefore hypothesized that the *P. aeruginosa* AR1 and AR4 SLiMs, along with the newly identified REER-repeat region, might be involved in RNA binding. To test this hypothesis, we conducted electrophoretic mobility shift assays (EMSA) to examine the binding of purified RNase E CTD proteins (shown in S6A Fig) to an *in vitro* synthesized RNA derived from the *malEF* intergenic region (for details, see Materials and methods). We tested wild-type (WT) CTD and mutated variants where arginine residues in AR1, AR4, or the REER-repeats were replaced with alanine (named AR1mut, AR4mut, and REERmut, respectively; see S4 Table for a description of the mutations). Qualitative analysis of the EMSA showed that the WT, AR1mut, and AR4mut variants bound RNA with similar efficiency, as indicated by comparable gel shift patterns (Figs 2A and S4A). However, the REERmut variant exhibited slightly reduced RNA binding, evidenced by a higher concentration requirement for mobility shift as compared to the former proteins (Figs 2A and S4B). The combination of AR1 and AR4 mutations resulted in an impaired RNA binding which was further exacerbated when the REER mutation was also introduced: no observable shift in the RNA was detected when incubated with the protein variant carrying mutations in all three SLiMs, even at an RNA-to-protein ratio of 1:80 (Fig 2A). Together, these results highlight the functional redundancy of the AR1, AR4, and REER-repeat SLiMs in RNA binding, as a complete defect in RNA binding is observed only when all these SLiMs are simultaneously disrupted. Additionally, their potential cooperativity may enhance RNA binding affinity for specific targets.

One notable characteristic of the RNA degradosome is its tendency to form highly dynamic subcellular foci, also referred to as “clusters,” “puncta,” or “helical structures” [27,62]. These foci rapidly disassemble upon rifampicin treatment, suggesting their formation is RNA-dependent [62,63]. In *E. coli*, these foci are membrane-anchored via the MTS SLiM, whereas in *C. crescentus* they remain cytoplasmic, driven by liquid-liquid phase separation [63,64]. To investigate the subcellular localisation of Pa RNase E, we constructed *P. aeruginosa* PAO1 strains expressing msfGFP-tagged Pa RNase E, Pa RNase E^1-588^, or Pa RNase E^1-529^ from the native *rne* locus. The two msfGFP-tagged RNase E truncations, whose stability was confirmed by Western Blot (S5A Fig), were designed based on our in-silico analysis of Pa RNase E SLiMs, assuming that they would lack the capacity to assemble the RNA degradosome. The key difference between RNase E^1-588^ and RNase E^1-529^ lies in the presence of the MTS (Fig 1B). Visualization using epifluorescence microscopy revealed that RNase E-msfGFP forms foci concentrated near the cell edge (Fig 2B). Both CTD truncation mutants showed impairment in foci assembly, but while the RNase E^1-588^-msfGFP protein localises uniformly around the edge of the bacteria, the RNase E^1-529^-msfGFP mutant protein additionally fails to localise near the membrane, consistent with the role of the MTS in membrane anchoring (Fig 2B-2C).

Exposure of Pa RNase E-msfGFP-expressing cells to high levels of rifampicin (100 µg/ml) led to the dissipation of these foci, resulting in a smoother distribution of the signal along the cell periphery, which phenocopies the localization of the RNase E^1-588^-msfGFP strain (Fig 2D). Quantitative analysis confirmed a significant reduction in signal intensity variance upon rifampicin treatment as compared to the control (DMSO-only), indicating foci dissipation, while the average signal intensity remained constant, suggesting that RNase E-msfGFP is stable during rifampicin exposure (Figs 2E, 2F, and S4F). The eventuality of RNase E-msfGFP proteolysis was indeed ruled out by Western Blot (S5B Fig).

To further verify that Pa RNase E-msfGFP foci formation depends on CTD-mediated RNA binding, we constructed *P. aeruginosa* strains with chromosomally msfGFP-tagged RNase E variants mutated in AR1, AR4, and REER-repeats SLiMs, individually and in combination, as described previously for the EMSA. Visualisation of these variants showed a clear correlation between CTD-mediated RNA binding and foci formation. Specifically, the RNase E AR1+AR4mut-msfGFP showed less distinct foci and lower signal variance compared to RNase E-msfGFP and single AR1 or AR4 mutants (Fig 2B and 2C). The RNase E REERmut-msfGFP also had diffuse foci and lower variance, while the RNase E AR1+AR4+REERmut-msfGFP, with abolished CTD-mediated RNA binding, displayed the most severe disruption, with nearly complete loss of foci and a very narrow signal intensity distribution (Figs 2B, 2C, and S4F). Similar patterns were observed with four other CTD truncations containing different combinations of RNA-binding SLiMs, ruling out the possibility that this foci dissipation is caused by the changes in the charge of the CTD conferred by the Arg to Ala mutations (S4E Fig).

Of note, we noticed an increased signal intensity in all the msfGFP-tagged RNase E mutants tested as compared to the RNase E-msfGFP (S4C Fig). Since all constructs are expressed at the native locus, this could likely reflect higher RNase E protein levels, which suggests that CTD-mediated RNA binding might be necessary for RNase E autoregulation [65]. To confirm this, we performed a Western Blot on strains expressing several Strep-tagged or msfGFP-tagged RNase E mutants and could indeed observe that RNase E protein levels are increased upon deletion of the CTD or mutation of AR1+AR4+REER SLiMs (S4D and S5 Figs).

Overall, we conclude that in *P. aeruginosa*, the organisation of the RNA degradosome complex into visualisable foci is dependent on the RNase E CTD-mediated RNA binding and suggest that this interaction not only organizes the localisation of the RNA degradosome but could also influence the regulatory activity of RNase E.

### Identification of proteins binding to RNase E scaffold domain

**Fig 3:**
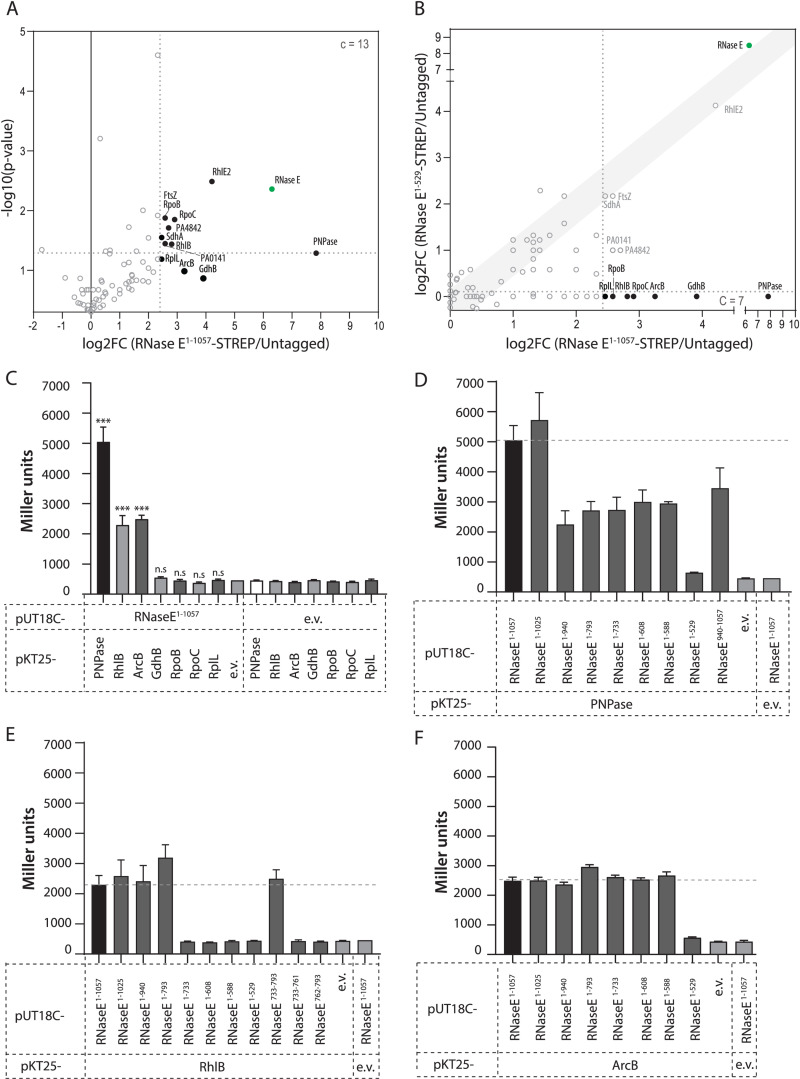
Identification of scaffold domain-dependent RNase E protein partners by pull-down assays and mapping of the interactions by bacterial two-hybrid assays. **(A)** Volcano plot highlighting the relative enrichment of proteins co-eluting with the RNase E^1-1057^-Strep bait protein compared to the untagged negative control. The plot was generated from the average peptide count obtained in duplicate pull-down experiments. An arbitrary threshold of 5.5-fold change was chosen for selection of candidate (c) proteins. **(B)** Scatterplot highlighting comparative enrichment of proteins co-eluting with the RNase E^1-1057^-Strep bait protein (x axis) or with the RNase E^1-529^-Strep bait protein (y axis) compared to the untagged negative control. **(C)** Quantification of β-galactosidase activity in *E. coli* BTH101 cells co-expressing T18-RNase E with T25-PNPase, T25-RhlB, T25-ArcB, T25-GdhB, T25-RpoB, T25-RpoC, or T25-RplL. Interaction between T18-RNase E and a T25-tagged protein partner results in a significant restoration of β-galactosidase activity compared with empty vector (e.v.) negative controls. Unpaired Welch’s t-tests were performed using GraphPad Prism to assess differences between every tested protein-protein interaction and the respective negative control. ***: p-value <0.0001; n.s: not significant (99% confidence level). **(D-F)** Mapping of the interaction between RNase E and PNPase **(D)**, RhlB **(E)**, or ArcB **(F)** by quantification of β-galactosidase activity in *E. coli* BTH101 cells co-expressing T25-PNPase **(D)**, T25-RhlB **(E)**, or T25-ArcB **(F)** and either T18-RNase E or one of eight to ten truncated T18-RNase E fragments. Empty vector (e.v.) negative controls are included into each separate graph for comparison.

In *P. aeruginosa*, the RNA degradosome was proposed to include the RhlE2 RNA helicase (PA0428), PNPase, and other associated proteins [53,54]. However, the direct nature of these interactions and the specific interaction sites have not been demonstrated nor mapped. Additionally, RNase R was also suggested to be a *P. aeruginosa* RNA degradosome component, as it is in *P. syringae* LZ4W [29,39]. To test these hypotheses and identify Pa RNase E CTD interacting proteins, we performed a pull-down assay using *P. aeruginosa* strains expressing C-terminal Strep II-tagged full-length RNase E (RNase E^1-1057^-Strep) or an RNase E truncation missing the entire CTD (RNase E^1-529^-Strep). As an additional control, the assay was also performed with the *P. aeruginosa* PAO1 wild-type (WT) to discard proteins interacting nonspecifically with the Strep-Tactin magnetic beads (Fig 3A and 3B).

Eluates of the pull-down experiment were first analysed by silver staining (S6B Fig). The RNase E^1-1057^-Strep eluate revealed a high molecular weight band corresponding to RNase E, along with additional bands absent in the untagged and RNase E^1-529^-Strep samples. These bands likely represent components of the RNA degradosome or cleaved RNase E^1-1057^-Strep intermediates, as RNase E is known to be prone to cleavage [41,66]. Mass spectrometry (LC-MS/MS) analysis identified a total of 115 proteins present in both duplicates of the RNase E^1-1057^-Strep eluates (S5 Table). 13 proteins (including RNase E) were enriched by at least 5.5-fold as compared with the untagged sample (Fig 3A). This stringent threshold was chosen since core components of the RNA degradosome should be abundantly associated to RNase E. Of these 12 putative partners, 5 (PNPase, GdhB, ArcB, RhlB and RpoB) were completely absent from both RNase E^1-529^-Strep duplicates and 2, namely RpoC and RplL, were absent from one replicate and present in the second replicate with a peptide count of 2 (Fig 3B and S5 Table). We selected for further study these 7 proteins, which were considered the most promising RNA degradosome candidates as their interaction with Pa RNase E seemed dependent on the CTD.

It is worth noting that the RhlE2 RNA helicase was, as expected [53,54], among the proteins enriched in the RNase E^1-1057^-Strep eluate as compared to the untagged control, but surprisingly it was equally enriched in the RNase E^1-529^-Strep eluate condition (Fig 3B), indicating that this interaction occurs via RNase E NTD and not via the CTD.

### RNase E direct interaction with PNPase and RhlB occurs via the NDPR and AR1 SLiMs, respectively

The interaction between RNase E and the 7 candidates was first verified using an adenylate-cyclase-based bacterial two-hybrid (BTH) assay [67]. Co-expression of T25-PNPase and T25-RhlB with T18-RNase E resulted in red colonies on MacConkey agar and a significant increase of β-galactosidase activity compared to empty vector controls (11-fold or 5-fold increase, respectively), confirming their interaction (Figs 3C and S7A). Co-expression of T25-ArcB with T18-RNase E also resulted in a significant increase in β-galactosidase activity (5-fold increase) compared to the negative control (Figs 3C and S7A). To identify the SLiMs mediating the observed interactions, we generated seven CTD truncations of RNase E, each lacking an additional SLiM as compared with the longer version. Testing these truncations for their capacity to interact with PNPase revealed that only T18-RNase E^1-1025^ showed a β-galactosidase activity similar to that of T18-RNase E^1-1057^ upon co-expression with T25-PNPase (Figs 3D and S7B). In contrast, T18-RNase E^940-1057^ and all shorter T18-RNase E CTD truncations except T18-RNase E^1-529^ exhibited a significant increase of β-galactosidase activity as compared with the negative controls but lower than T18-RNase E^1-1057^ (Figs 3D and S7B). Overall, this suggests that PNPase interaction with RNase E relies on a complex binding site involving RNase E residues 529-588 and 940-1057.

Co-expression of the T18-RNase E CTD truncations with T25-RhlB revealed that the protein-protein interaction is mediated by residues 733-793: no significant interaction could be seen between T25-RhlB and T18-RNase E^1-733^, although T25-RhlB could interact with T18-RNase E^1-793^ (and every longer CTD truncation) or with T18-RNase E^733-793^ about as strongly as with T18-RNase E^1-1057^ (Figs 3E and S7C). Of note, neither T18-RNase E^733-761^ nor T18-RNase E^762-793^ were able to restore β-galactosidase activity above negative control levels when co-expressed with T25-RhlB (Fig 3E), suggesting that the entire AR1 SLiM, as defined in Fig 1B, is necessary for interaction with RhlB.

Surprisingly, all T18-RNase E CTD truncations, except T18-RNase E^1-529^, significantly interact with T25-ArcB, suggesting a complex binding that is dependent on residues 529-588 (Figs 3F and S7D). Of note, these residues only contain the MTS SLiM, which is presumably involved in membrane binding.

**Fig 4:**
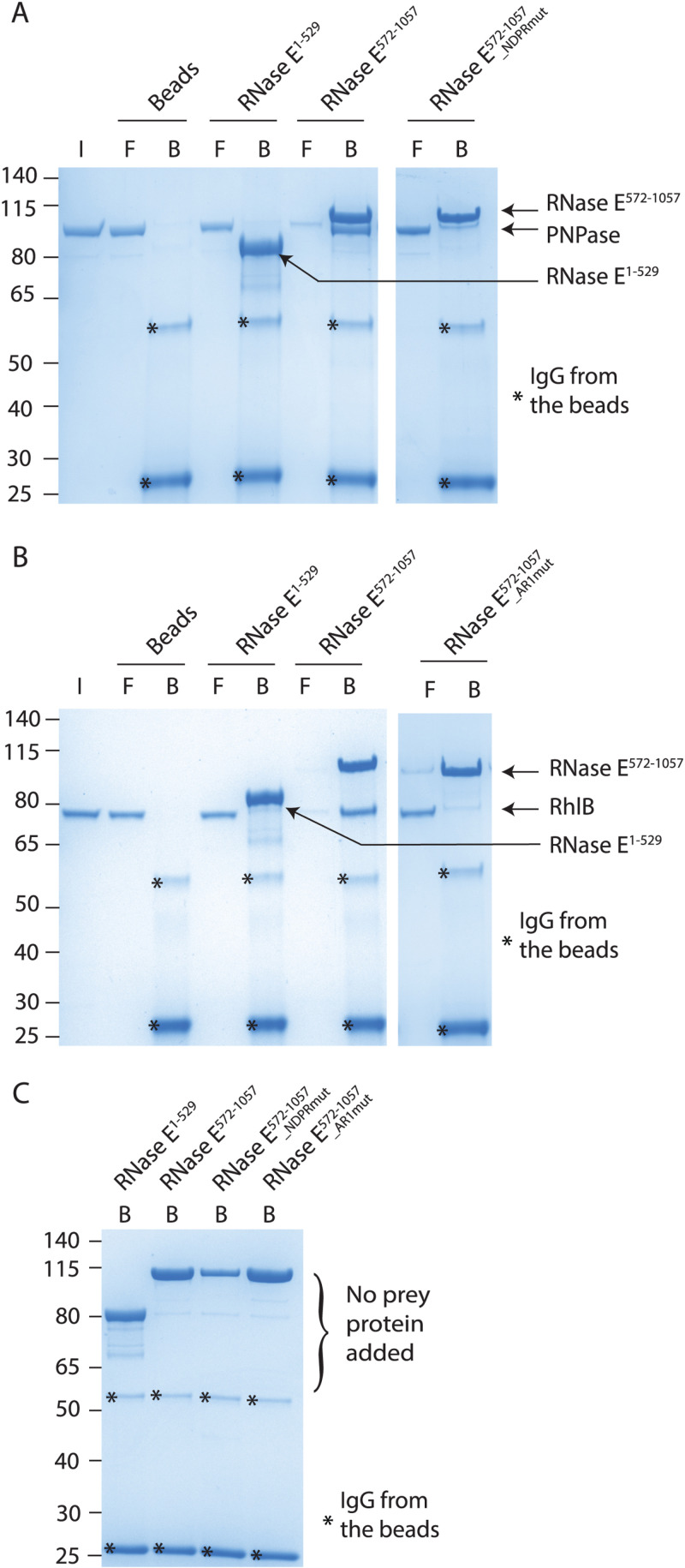
PNPase and RhlB directly interact with RNase E scaffold domain via NDPR and AR1 SLiMs, respectively. Purified (His_10_-Smt3) Flag-tagged RNase E^1-529^ or RNase E^572-1057^ proteins having either native or mutated sequence were used as baits, immobilized to anti-flag magnetic beads and incubated with purified His_10_-Smt3 tagged PNPase **(A)**, RhlB **(B)**, or buffer only **(C)** to assess direct protein binding. I: input, F: free fraction, B: bound fraction. NDPRmut, AR1mut: mutations of NDPR and AR1 SLiMs, respectively (see S4 Table). Binding assays were repeated at least 2 times and representative gels are shown.

Building on the bacterial two-hybrid assays results, we successively employed affinity chromatography with purified proteins to investigate direct binding, minimizing the influence of other protein partners or RNA. C-terminal 3xFlag tagged RNase E^1-529^ and RNase E^572-1057^ (CTD with no MTS) were purified and immobilized on anti-Flag magnetic beads. After extensive washing, the beads were mixed with either purified proteins (PNPase or RhlB) or buffer only, and the free or bound fractions were analysed by SDS-PAGE (Fig 4).

PNPase was retained on beads containing Flag-RNase E^572-1057^, but not on beads alone or containing Flag-RNase E^1-529^ (Fig 4A), indicating that the RNase E CTD is sufficient for direct interaction with PNPase. Point mutations within the NDPR SLiM (Flag-RNase E^572-1057^_NDPRmut) abolished the binding, indicating that this SLiM is necessary for direct interaction (Fig 4A). Of note, PNPase is not retained on beads containing Flag-RNase E^1-588^, which suggests that residues 529-588 are not sufficient to mediate a direct protein-protein interaction, with the NDPR SLiM being the major binding site (S6C Fig). Similarly, RhlB was retained on Flag-RNase E^572-1057^ but not on Flag-RNase E^1-529^ and point mutations in the AR1 SLiM (Flag-RNase E^572-1057_^AR1mut) showed that this region is crucial for RhlB binding (Fig 4B).

Contrastingly, ArcB did not clearly co-elute with Flag-RNase E^1-1057^ or Flag-RNase E^1-588^ under the tested conditions, suggesting an indirect interaction (S6D Fig).

To summarize, these experiments clearly establish PNPase and RhlB as core components of *P. aeruginosa* RNA degradosome and demonstrate that the NDPR and AR1 SLiMs are essential for their direct interactions with RNase E.

### PNPase and RhlB are dependent on their physical interaction with RNase E for colocalising in RNA degradosome foci

**Fig 5:**
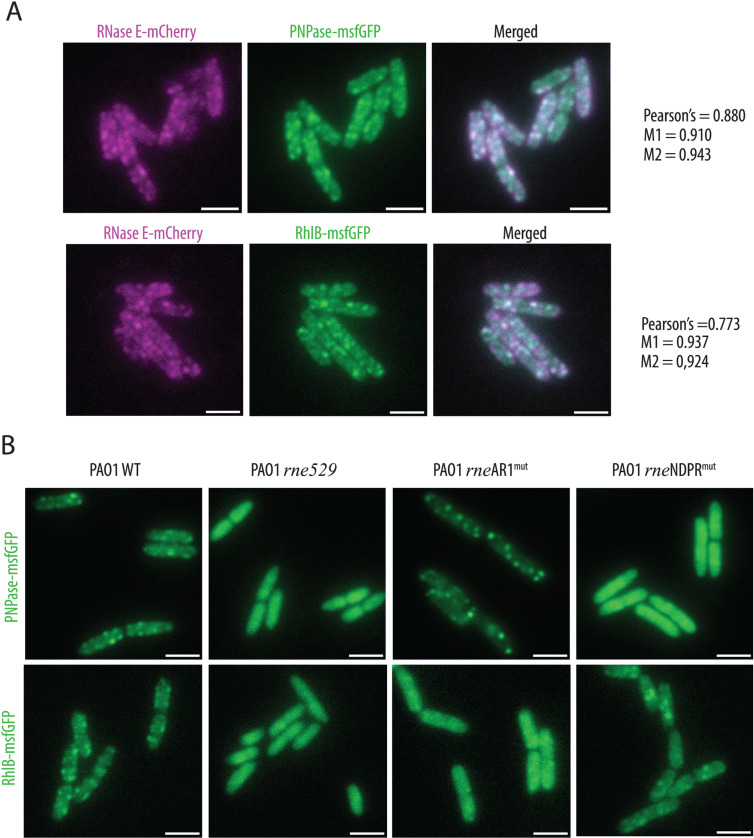
Colocalisation of PNPase or RhlB with RNase E foci is dependent on their interaction with NDPR and AR1 RNase E SLiMs, respectively. **(A)** Visualisation of PNPase-msfGFP (upper panels) or RhlB-msfGFP (lower panels) fusion and RNase E-mCherry fusion co-expressed in the same strain. Pearson’s and M coefficients were calculated in Fiji using the JaCoP plugin [68]. Three independent images were used, each containing at least 30 cells, and an average coefficient was calculated from the three values obtained with each image. **(B)** Visualisation of PNPase-msfGFP (upper panels) and RhlB-msfGFP (lower panels) in strains expressing either the native RNase E protein (WT) or truncated variants, as indicated above each image. Scale is 2 μm.

Given the foci localization of RNase E, we assessed PNPase and RhlB colocalization with RNase E by using *P. aeruginosa* strains carrying *rne::mCherry* and either *pnp::msfGFP* or *rhl::msfGFP* chromosomal fusions. As expected, both PNPase-msfGFP and RhlB-msfGFP formed foci near the membrane and co-localized with RNase E-mCherry in live cells (Fig 5A). Overlap coefficients between the two fluorescence channels were consistently over 0.9, while Pearson’s coefficients were slightly lower (0.880 for PNPase and 0.773 for RhlB), likely due to differences in signal intensity. Of note, strains expressing any of these chromosomal fusion proteins grow identically to the WT strain at 37°C, suggesting that all the fusion proteins are functional or at least not detrimental to growth (S8A Fig). We also assessed the subcellular localisation of ArcB-msfGFP and observed a smooth distribution rather than foci, which suggests that the interaction between RNase E and ArcB is likely transient, with ArcB not being a core component of the complex (S8C Fig). Alternatively, it is possible that only a small fraction of the cellular ArcB pool is bound to RNase E, rendering it undetectable when examining the overall localization of ArcB.

To further corroborate the need for specific RNase E domains in these interactions, we examined the localization of PNPase-msfGFP and RhlB-msfGFP in strains expressing RNase E^1-1057^ (WT), RNase E^1-529^ (*rne529*), RNase E^AR1mut^ (*rne*AR1^mut^), or RNase E^NDPRmut^ (*rne*NDPR^mut^). Both PNPase-msfGFP and RhlB-msfGFP displayed diffuse cytosolic localization in the *rne529* strain compared to the WT strain (Fig 5B). In the *rne*NDPR^mut^ strain, PNPase-msfGFP showed a diffuse cytosolic distribution, while RhlB-msfGFP was diffuse in the *rne*AR1^mut^ strain, highlighting an impaired recruitment into the RNase E foci. However, the NDPR or AR1 mutations did not prevent the other protein partner from being recruited into the foci, indicating that both proteins can be independently recruited into RNase E-driven foci (Fig 5B). We also confirmed that RNase E NDPR^mut^-msfGFP forms foci, ruling out the possibility that the loss of PNPase foci in the *rne*NDPR^mut^ strain is due to impairment of RNase E-driven foci formation (S8B Fig). These findings confirm the *in vivo* interaction of Pa PNPase and Pa RhlB with the NDPR and AR1 SLiMs, respectively, and demonstrate that these interactions are crucial for their near-membrane foci localization.

### Phenotypic changes associated with partial or complete deletion of RNase E CTD

To investigate the biological importance of RNase E CTD-mediated complex scaffolding in *P. aeruginosa*, we performed some phenotypic assays using the *rne529* and *rne588* strains, as well as strains bearing the AR1 mutation (*rne*AR1^mut^), NDPR mutation (*rne*NDPR^mut^), or AR1+AR4+REER mutation (*rne*AR1+AR4+REER^mut^), affecting RhlB binding, PNPase binding, or CTD-mediated RNA binding plus RhlB binding, respectively.

**Fig 6:**
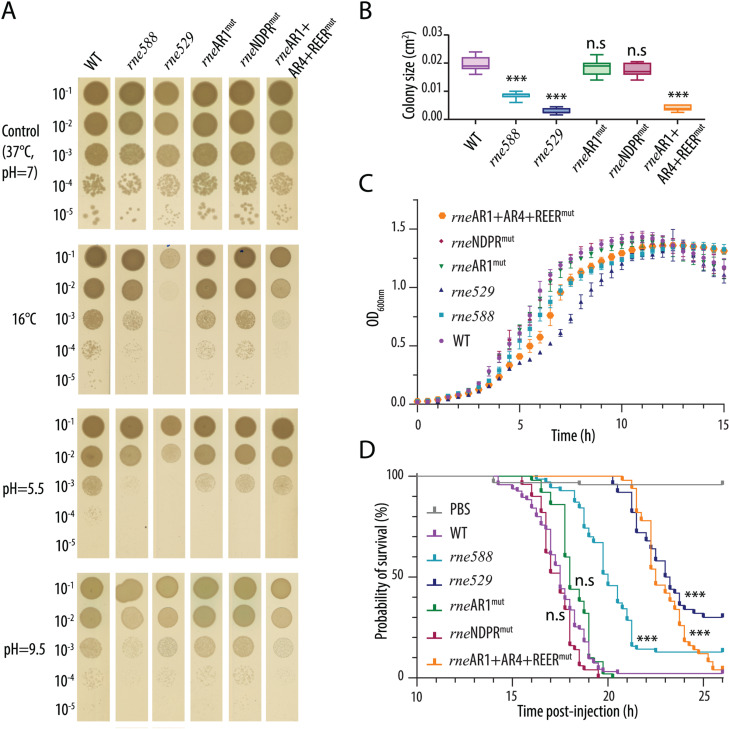
Phenotypic changes caused by deletion or mutation of RNase E scaffolding domain. **(A)** Growth on NA agar plates at 37°C or 16°C, or under low vs high pH conditions of wild-type (WT), *rne588*, *rne529*, *rne*AR1^mut^, *rne*NDPR^mut^, and *rne*AR1+AR4+REER^mut^ strains, as indicated. Experiments were performed in biological independent triplicates; a representative plate is shown. **(B)** Measured colony size for the different mutant strains as indicated. Quantification was done using the Measure function in Image J after drawing a circle around the edge of each colony. 11 to 13 colonies were quantified for each strain. A one-way Anova test was performed using GraphPad and comparing the colony size of each mutant strain to that of the WT strain. ***: p-value <0.0001; n.s: not significant (99% confidence interval). **(C)** Growth in NYB broth at 37°C in 96-well plates of wild-type (WT), *rne588*, *rne529*, *rne*AR1^mut^, *rne*NDPR^mut^, and *rne*AR1+AR4+REER^mut^ strains, as indicated. Independent biological triplicates, each with four technical replicates, were used to generate the average growth curve shown. **(D)** Killing curves of *G. mellonella* larvae injected with 10 μL of WT or mutant *P. aeruginosa* strains (described above). The control group was injected with 10 μL of sterile PBS instead. Larvae were incubated at 37°C and larval death was monitored over 13-26 hours post-injection. A larva was considered dead when it completely stopped responding to repeated touching stimuli. At least two independent replicates were performed using a total of at least 50 larvae for each strain tested. A Kaplan-Meier test for survival analysis was performed using GraphPad Prism and comparing the survival curve of each mutant strain to that of the WT strain. ***: p-value <0.0001; n.s: not significant (99% confidence interval).

On solid media at 37°C, the *rne529*, *rne588* and *rne*AR1+AR4+REER^mut^ strains displayed no severe growth defect but a significantly reduced colony size, suggesting a growth delay and/or increased cell death (Fig 6A top panel, Fig 6B). The *rne529, rne588* and *rne*AR1+AR4+REER^mut^ strains were also slightly but consistently affected at 37°C in liquid culture (Fig 6C). To verify that this was caused by truncations/mutations introduced in the *rne* gene, we electroporated the WT or the two most impaired mutant strains with an inducible vector allowing in-trans expression of RNase E, RNase E-msfGFP, or RNase E-mCherry. Cultivation of the strains at 37°C in liquid culture revealed that expression of either native or fluorescently-tagged RNase E could restore identical growth levels compared to the electroporated WT strain, additionally confirming that the fluorescently-tagged RNase E protein is fully functional (S9 Fig).

Interestingly, when cultured on solid media at 16°C, the growth defects of the *rne529*, *rne588* and *rne*AR1+AR4+REER^mut^ strains became more pronounced, with *rne529* barely growing (Fig 6A, upper middle panel). Since the *rne*AR1^mut^ and the *rne*NDPR^mut^ strains exhibited no significant differences compared to the WT, these results indicate that the cold sensitivity of the *rne588* strain can be attributed to the loss of RNase E CTD RNA binding. Moreover, the severe growth defect observed in the *rne529* mutant indicates that additional delocalisation of RNase E from the membrane exacerbates the cold sensitivity caused by deletion of RNase E RNA-binding SLiMs.

Besides low temperature, we also tested sensitivity to low or high pH (Fig 6A, lower panels): while growth at pH≃9.5 is rather similar between the different strains, deletion of the CTD (*rne529* and *rne588*) impairs growth at pH≃5.5, although the three CTD point mutant strains are mostly unaffected. This suggests that, although loss of interaction with PNPase or RhlB separately does not seem to impair growth, the combined loss of RNase E protein-protein and protein-RNA interactions results in a growth defect under low pH conditions. Finally, we assessed whether the RNase E CTD mutants might be impaired in virulence using *Galleria mellonella* larvae, a highly predictive infection model for studying mammalian infection processes [69]. Infection with PAO1 WT, *rne*AR1^mut^ and *rne*NDPR^mut^ strains resulted in nearly 100% larval mortality within 20 hours post-injection (Fig 6D). In contrast, the larvae infected with the *rne588* mutant exhibited 50% survival at this time point, while those infected with *rne*AR1+AR4+REER^mut^ or *rne529* mutants showed 100% survival, with larval death occurring only after 21 hours post-injection (Fig 6D). These results strongly indicate that the ability of *P. aeruginosa* to infect a host is compromised when RNase E CTD-mediated RNA binding is disrupted. This impairment is likely attributable to several contributing elements: reduced bacterial growth within the host, deficiencies in virulence factor production, and a decreased ability to endure host immune responses and host-related stresses.

### Effect of RNase E CTD on *P. aeruginosa* transcripts steady-state levels

**Fig 7:**
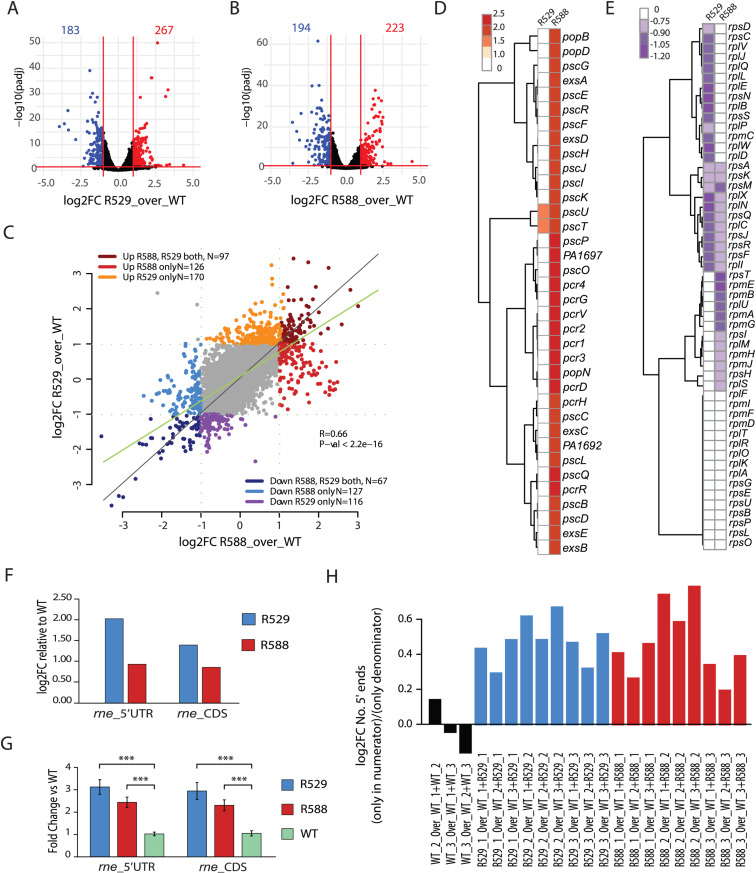
RNA-seq analysis of RNase E CTD truncated mutants reveals broad transcriptome changes. **(A-B)** Volcano plots highlighting genes differentially regulated in the **(A)** *rne529* (R529) or **(B)** *rne588* (R588) mutant strains compared with the WT strain. Genes significantly up- or down- regulated (adjusted p-value < 0.05, fold change |FC| ≥ 2) are highlighted in red or blue, respectively. **(C)** Scatterplot of genes differentially regulated in R529 strain versus genes differentially regulated in R588 strain, highlighting commonalities and differences between the two mutant strains. **(D-E)** Clustered heat maps of type 3 secretion system **(D)** or ribosomal proteins **(E)** transcript levels in the R529 and R588 mutants. **(F)** Differential analyses (log_2_-fold change) of normalized total reads counts mapping to the 5’ untranslated region of the *rne* transcript (*rne*_5’UTR) or the coding sequence of the *rne* N-terminal domain (*rne*_CDS) in the R529 and R588 mutants as compared to the WT strain. **(G)** RT-qPCR analysis of relative expression levels (fold change versus WT) with standard error of *rne*_5’UTR or *rne*_CDS in the R529 and R588 mutants. Expression levels were normalised for each sample using *rpoD* as an internal control. ***: p-value <0.001. See Materials and methods for details. **(H)** Proportion of loci with 5’ read ends (sequenced fragments) counted only in a strain (numerator) relative to the 5’ read ends counted in that strain and in a WT replica (denominator). A total of 8 million reads was analysed in each strain to exclude an effect due to the library depth (see Materials and methods). Histograms of individual values calculated per each strain and replicate is shown in S11 Fig.

To better understand the phenotypes observed and evaluate the impact of RNase E CTD loss on *P. aeruginosa* transcriptome, we conducted RNA-sequencing analysis on *P. aeruginosa* WT, *rne529,* and *rne588* mutant strains grown at 37°C until the late-exponential growth phase (OD_600nm_ of 1-1.2). Biological triplicates were analysed and show a high degree of relatedness within each strain (S10 Fig). Under these conditions, the loss of the entire RNase E CTD in the *rne529* strain affected the steady-state levels of 450 transcripts, with 183 downregulated and 267 upregulated (adjusted p-value < 0.05, fold change |FC| ≥ 2) (Fig 7A). In the *rne588* mutant, 417 transcripts were differentially expressed, with 194 downregulated and 223 upregulated (adjusted p-value < 0.05, fold change |FC| ≥ 2) (Fig 7B). Differential expression analysis on reads mapping to predicted small RNA genes (sRNAs) identified 14 predicted sRNA genes with significantly enriched reads in the *rne529* mutant and 5 in the *rne588* strain (S6 Table). Altogether, these changes represent both direct and indirect effects of altered RNase E cleavage. A comparison of the *rne529* and *rne588* strains revealed that 164 mRNAs were consistently regulated in both mutants (97 upregulated and 67 downregulated, see Fig 7C). Among the strongest commonly downregulated genes were: (i) the *pqsABCD* genes, involved in the synthesis of quorum-sensing signal molecules alkylquinolones PQS (2-heptyl-3-hydroxy-4(1H)-quinolone) and HHQ (2-heptyl-4(1H)-quinolone), which play roles in virulence [70], (ii) the *phzA1B1* and *phzA2B2* phenazine biosynthesis genes, which are important for synthesis of the toxic secondary metabolites phenazine and pyocyanin [71], and (iii) the *agtABCD* gene cluster for 4-aminobutyrate (GABA) and 5-aminovalerate (AMV) uptake [72]. Surprisingly, the two strains exhibited more differences in mRNA levels than shared changes. In the *rne588* strain, the mRNA levels corresponding to type 3 secretion system genes, which deliver toxins to host cells [73], were consistently upregulated, while they did not vary significantly in the *rne529* strain as compared to the WT (Fig 7D). On the other hand, ribosomal and many amino acid metabolism genes (tryptophan, arginine, alanine, aspartate and glutamate) were downregulated only in the *rne529* strain, which could correlate with the general growth defect observed (Fig 7E and S6 Table).

Considering the autoregulation of RNase E reported in *E. coli* and *C. crescentus* [65,74], we quantified reads mapping to the 5’ untranslated region (UTR) of the *rne* gene (transcription start site determined in [75]) and observed increases of 2 and 0.96 log_2_ folds in the *rne529* and *rne588* mutants, respectively, compared to the WT (Fig 7F). Similarly, we observed a 1.43 and 0.88 log_2_ fold increase in the *rne* coding sequence (CDS) corresponding to the NTD of RNase E in the *rne529* and *rne588* mutants, respectively (Fig 7F), in agreement with the observed increase in protein levels (S4C, S4D and S5A Figs). We validated these data in a RT-qPCR experiment, which revealed a similar increase in relative *rne* 5’UTR and *rne* CDS expression levels for the *rne529* and *rne588* mutants (Fig 7G). Finally, to assess RNA degradation efficiency in our strains, we analysed the 5’ reads ends of sequenced RNA fragments. As previously performed in other studies [55,76], we calculated the ratio between the number of loci with 5’ ends present only in a mutant and not in the WT, over the sum of loci with 5’ read ends seen in both mutant and WT. We performed the analysis in the three genetic backgrounds (WT, *rne529*, *rne588*) and for each replica independently to ensure consistency (see Materials and methods and S11 Fig). Despite the background of random fragmentation intrinsic to the sequencing protocol, a consistent increase on the number of 5’ ends in the *rne529* and *rne588* strains was observed as compared to the WT (Fig 7H), suggesting an accumulation of RNA degradation intermediates and an altered RNA degradation in the RNase E truncated mutants.

## Discussion

The composition and the functions of the RNA degradosome vary among bacterial species, likely reflecting diverse lifestyles and niche adaptations [29]. Studying the RNA degradosome in various bacterial species is therefore crucial for elucidating how bacteria respond and adapt to different environmental conditions and sustain various types of stress. In this study, we characterized the RNA degradosome of *P. aeruginosa*, a versatile opportunistic pathogen and widely used model organism for bacterial pathogenesis and environmental adaptation.

**Fig 8:**
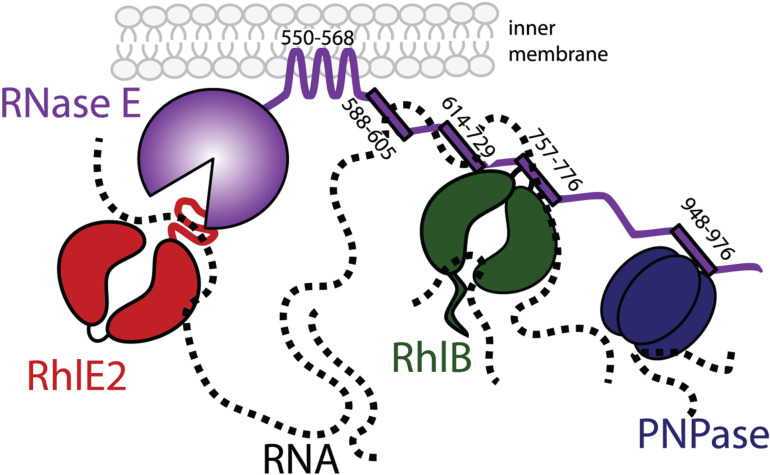
Model of the RNA degradosome in *P. aeruginosa.* Numbers corresponds to RNase E SLiM start-end residues involved in membrane, RNA, RhlB or PNPase interaction. RNA is represented as a dotted line. See discussion for details.

In the first part of our study, we focused on the putative SLiMs within the CTD of *P. aeruginosa* RNase E and assessed their contribution to RNA binding and foci assembly. We successively identified RhlB and PNPase as core RNA degradosome components, as well as the SLiMs mediating these interactions (Fig 8). A notable feature of SLiMs is their poor sequence conservation, even when they mediate interactions with the same protein partner. Our findings confirm this variability for both PNPase and RhlB interactions.

PNPase, a known partner of RNase E in several species, binds through SLiMs with significantly different sequences across organisms. For instance, in *Anabaena* PCC7120, the interaction occurs via a cyanobacterial SLiM having the conserved residues RRRRRRSSA [36], while in *E. coli* or *C. crescentus*, the sequences are WQRPTFAFEGKGAAGGHTATHHASA [29] and APPEKPRRGWWRR [42], respectively. Our pull-down assays and *in vitro* binding assays showed that PNPase interacts with RNase E in *P. aeruginosa* via the NDPR SLiM, which has yet another distinct sequence (TGRALNDPREKRRLQREAERLAREAAAAA). These sequence variations may reflect species-specific interactions, as suggested by experimental work showing lack of heterologous interactions between RNase E and PNPase of *E. coli* and *Pseudoalteromonas haloplanktis* [41]. Despite sequence variability, the PNPase interacting SLiM is consistently located towards the end of the RNase E CTD [29,36,42], suggesting that the positioning of the PNPase relative to other RNA degradosome components and to RNase E catalytic domain is constrained.

Of note, a recent study on a *P. aeruginosa* mutant with a 50 bp deletion in the 3’ end of the *rne* gene revealed a hypervirulence phenotype, characterized by enhanced cytotoxicity and siderophore production [77]. Based on *E. coli* RNA degradosome model, this phenotype was associated with a possible impairment of PNPase binding to Pa RNase E or a different RNA degradosome activity [77]. Our data reveal that PNPase can interact with the RNase E^1-1025^ truncation (BTH assay), ruling out the former hypothesis. However, we cannot exclude the possibility that this region might be involved in interaction with a yet unknown partner.

Regarding RhlB, we show that in *P. aeruginosa*, this RNA helicase directly interacts with RNase E via the AR1 SLiM (RPRRRSRGQRRRSNRRERQR). Mutation of AR1 disrupts not only the RhlB-RNase E binding *in vitro*, but also RhlB subcellular localisation near the membrane and co-localization with RNase E *in vivo*. Interestingly, in *E. coli* and other species the AR1 SLiM mediates RNA interaction [29,41]. In *P. aeruginosa*, AR1 also contributes to RNA-binding in addition to RhlB binding. Given that in *E. coli,* a single arginine mutation within the HBS SLiM (R730A) is sufficient to disrupt RhlB binding [78], it is possible that only one or a few arginine residues of the AR1 SLiM are involved in interaction with RhlB, while the others mediate RNA binding. Further experiments are needed to address this hypothesis and to understand the dynamics of the RhlB-RNA-RNase E complex in *P. aeruginosa*. Moreover, it has been shown that the RNA-dependent ATPase and unwinding activity of RhlB are stimulated by binding to RNase E [79]. Future studies will investigate whether it is also the case in *P. aeruginosa*.

In contrast with the *E. coli* RNA degradosome, we did not find any evidence of an interaction between RNase E and enolase in *P. aeruginosa* when the bacteria grow in rich medium and standard laboratory conditions. Although our pull-down and bacterial-two hybrid data indicate a probable interaction between RNase E and ArcB, a catabolic ornithine carbamoyltransferase from the arginine deaminase pathway [80], no direct protein-protein binding could be observed *in vitro,* nor were the two proteins colocalising in vivo. We propose that RNase E indirectly interacts with ArcB. Indeed, the *P. aeruginosa arcDABC* polycistronic transcript undergoes endonucleolytic cleavage at multiple sites for its processing [81], suggesting that a regulatory interaction between RNase E and ArcB polypeptide could occur during co-translational RNA cleavage [17].

Additionally, the RNase E-based RNA degradosome of *P. haloplanktis, P. syringae*, and *R. capsulatus* do not seem to contain any metabolic enzyme under standard growing conditions [39–41]. This lack of RNase E interaction with metabolic enzymes may reflect distinct physiological roles of the RNA degradosome in different bacterial species, potentially linked to specific metabolic adaptations or regulatory mechanisms that modulate RNA stability and processing depending on nutrient availability.

However, we cannot exclude that some protein partners were missed as we chose a stringent threshold for analysis of the pull-down results. Transient or condition-specific accessory protein partners remain to be identified, and compositional biases such as the conserved AEPV-rich part of the RNase E CTD are not yet understood, as no ligand or biological function has been assigned to this region even in well-studied RNase E homologs.

It is also important to mention that throughout this study, we chose to focus on core RNA degradosome components that are identified based on their binding to RNase E CTD, but we cannot exclude that some functional interactions occur with the NTD. For example, RhlB was found to interact with RNase E NTD in *C. crescentus* [35], while in *Anabaena* the RNA helicase CrhB, RNase II and enolase were shown to associate with RNase E via its NTD [37,38]. Similarly, we found that the *P. aeruginosa* RhlE2 RNA helicase, previously shown to associate with RNase E in an RNA-dependent manner [54], interacts with the N-terminal domain of Pa RNase E. Although RhlE2 is not considered a core RNA degradosome component based on our assumption of CTD-mediated interactions, this association remains functionally significant [54, 55].

In the second part of our study, we evaluated the importance of RNA degradosome assembly at both transcriptomic and phenotypic levels through an analysis of *P. aeruginosa* strains carrying RNase E CTD truncations and mutations. Comparing the *rne588* and *rne529* strains allows us to discuss the importance of RNA degradosome assembly and membrane attachment in *P. aeruginosa*. The two mutants exhibited varying degrees of sensitivity to cold growth, with *rne529* being the most affected, indicating that cold growth requires both RNase E membrane attachment and RNA degradosome assembly. Transcripts levels were also generally affected in the mutants at 37°C, as well as global RNA degradation, as shown in the 5’ read ends analysis. Decreased degradation of cleaved transcripts could lead to an upregulation of their levels. Therefore, direct and indirect effects remain to be distinguished, and those changes remain to be correlated with proteins levels. Measuring mRNA half-lives in the mutants could help clarify these points.

While we could not find any descriptions in the literature of phenotypes associated with the deletion of the *rhl* gene (encoding RhlB) in *P. aeruginosa*, the *pnp* gene (encoding PNPase) is essential for bacterial viability [82]. However, a strain with a deletion of PNPase RNA-binding domains KH and S1 is viable and exhibits reduced virulence due to decreased expression of virulence factors such as T3SS, the type VI secretion system (T6SS), and pili [82,83]. Furthermore, PNPase regulates antibiotic resistance by controlling the MexXY multidrug efflux pump and PrtR, a negative regulator of pyocin biosynthesis [84,85]. Surprisingly, we did not find any phenotypic changes associated to the loss of RhlB or PNPase binding to RNase E, questioning the importance of the RNA degradosome protein association for the complex functionality. Competition assays could be useful in identifying any fitness defects in our mutants, as done in *E. coli* for RNase E CTD truncations [18,64], or different phenotypes or stresses could be tested. Moreover, examining the subcellular localization and dynamics of the RNA degradosome under these conditions could provide further insights into how the machinery operates in response to stress.

On the other hand, our phenotypic analysis highlights the crucial role of CTD RNA binding for the functionality of the RNA degradosome complex. Mutations in the SLiMs responsible for RNA binding (AR1, AR4, and the REER-repeats) not only impair *in vivo* RNA degradosome foci assembly and/or stability but also lead to decreased *P. aeruginosa* virulence and reduced growth at both 37°C and cold temperatures. The effect of these mutations on foci formation mirrors the effect observed upon cell exposure to rifampicin, which decreases RNA levels in the cell, and aligns with previous studies showing that Ec and Cc RNase E homologs also assemble foci in a CTD-dependent and RNA-dependent manner [62–64]. Our data thus suggest that impairing foci formation has a major effect on the activity of the RNA degradosome in *P. aeruginosa*. Future studies will address the catalytic activity of this RNase E mutated variant *in vivo* and *in vitro*.

Overall, our study identified core RNA degradosome components in *P. aeruginosa* and underscores the role of this complex in bacterial pathogenesis and adaptation to environmental changes. By revealing *P. aeruginosa*-specific interactions and their functional significance, our findings provide valuable insights into the molecular mechanisms of adaptation in this pathogen and highlight the potential of the RNA degradosome as a drug target.

## Materials and methods

### Bacterial strains and growing conditions

A list of bacterial strains used can be found in S1 Table. Unless otherwise specified, *P. aeruginosa* PAO1 was grown at 37°C in NYB (25 g of Difco nutrient broth, 5g of Difco yeast agar per liter) with shaking at 180 rpm or on Nutrient Agar (NA) (40 g Oxoid blood agar base, 5 g Difco yeast agar per liter). *E. coli* strains were similarly cultivated in LB broth or on LB agar instead. When required, antibiotics were added to these media at the following concentrations: 100 µg/ml ampicillin, 50 μg/mL kanamycin, 20 μg/mL tetracycline and 10 µg/ml gentamicin for *E. coli*; and 50 μg/mL or 100 μg/mL tetracycline and 50 µg/ml gentamicin for *P. aeruginosa*.

### Construction of plasmids used in this study

Information about plasmid construction and primers used in this study can be found in S2 and S3 Tables, respectively. Plasmids pUT18C and pKT25 were generally cloned by restriction enzyme digestion using EcoRI-HF and BamHI-HF (NEB). When a gene to be inserted could be internally cleaved by EcoRI and/or BamHI, the enzymes MfeI and/or BglII, respectively, were chosen instead during primer design to allow ligation of the insert to the vector digested with EcoRI/BamHI thanks to compatible cohesive ends. For cloning of *gdhB* and *rpoC* into pKT25, NEBuilder HiFi DNA assembly was used instead, due to the presence of many restriction enzymes internal cleavage sites. Ligation reactions were carried our using T4 DNA ligase (Roche or NEB) at 16°C overnight, or at room temperature for 2-3 hours, following standard protocols. When cloning inserts into the suicide vectors pME3087 or pEXG2, the NEBuilder HiFi DNA assembly Master Mix (NEB) was used according to the manufacturer’s recommendations. To constructs RNase E CTD mutants, the DNA was synthesized by GeneArt (ThermoFisher Scientific) and cloned into appropriate vectors for protein purification or strain construction. A description of the introduced mutations can be found in S4 Table. To construct pME6032-based expression vectors, restriction enzyme digestion was carried out using EcoRI and KpnI for both vector and inserts.

Ligation mixtures or the assembly reactions were transformed into chemically competent *E. coli* DH5α cells by heat-shock, plating on appropriate selective LB agar medium. The resulting colonies were screened by PCR for the presence of the insert in the plasmid of interest. After plasmid extraction (Miniprep kit, Sigma-Aldrich/Merck), plasmids were verified by Sanger sequencing at Microsynth.

### Construction of *P. aeruginosa* mutant strains

To construct chromosomal gene truncations or insert a Strep-tag or fluorescent protein into the chromosome, in frame with a gene of interest, the suicide vectors pEXG2 [86] or pME3087 [87] were used. Briefly, approximately 500 bp regions located immediately upstream or downstream of the region to be edited, and, when applicable, the tag or fluorescent protein of interest, were amplified by PCR and inserted together into the vector backbone using NEBuilder HiFi DNA assembly. After sequencing verification of the constructed plasmid quality, the plasmids were introduced into *P. aeruginosa* PAO1 strain by triparental mating using the *E. coli* strain HB101 (pRK2013). After selection with the appropriate antibiotic (tetracycline for pME3087 or gentamicin for pEXG2), merodiploids were resolved as described in [88] for pME3087 or [86] for pEXG2, and strains with the desired insertion/deletion were identified by colony PCR.

### RNase E sequence analysis

Group-specific homologs of RNase E in *P. aeruginosa*, *P. fluorescens*, *P. protegens*, *P. putida* and *P. stutzeri* were retrieved from NCBI databases using blastp [89] with *P. aeruginosa* PAO1 RNase E as a query. Only sequences with query coverage > 0.5 were conserved; a maximum of 5000 sequences were retrieved and analysed (upper limit of the online NCBI blastp program). The number of RE [E/D]R motifs in each protein sequence was determined using the vcountPattern function from the “biostrings” R package [90]. The similarity of the protein sequences within a taxonomic group was determined using the conserv function (method=“similarity”) from the bio3D R package on a muscle alignment of the protein sequences [91,92].

### Electrophoretic motility shift assay

The *malEF* transcript [93] was synthesized with a T7 transcription kit (Invitrogen) on linear DNA template (172 bp) obtained by PCR with primers p253-p254 (see S3 Table). The electrophoretic mobility shift assay (EMSA) was performed following the protocol of [94]. Briefly, the reaction mixtures (15 μl) contained 35 nM of RNA, 50 mM Tris-HCl, 100 mM NaCl, 5 mM DTT, 1.0 units/μl RNasin (Promega), and His_10_Smt3-RNase E^572-1057^ (wild-type and mutated variants) as specified, with protein concentration expressed as a molar ratio of relative to RNA concentration (see Figs 2A, S4A and S4B). The reactions were incubated at 4 °C for 30 min before loading on 2% agarose gel and run at 150 V for 15 min. RNA bands were visualized by gel staining with 5x SYBR Gold (Invitrogen) in 20 ml 1x TBE (89 mM Tris, 89 mM boric acid 2 mM EDTA). The RNA and agarose concentration was optimized to obtain the highest signal-to-noise ratio, as described in the published protocol [94].

### Epifluorescence microscopy imaging

Phase contrast and fluorescence microscopy were performed on a Zeiss Axio Imager 2 equipped with an objective alpha Plan-Apochromat 100x/1,46 Oil Ph3 M27 and a ZEISS Axiocam 305 mono CCD camera. Bacteria were grown until OD_600nm_= 0.2-0.6 (approx. 2.5 hours) in 3 ml of NYB and were then placed on an agarose pad containing 1% agarose dissolved in 0.5X PBS. Images were processed with Fiji (NIH, USA) [95].

### Image analysis

All image treatments and analysis were performed using Fiji (NIH, USA) [95]. Representative images shown had contrast adjusted for display by opening the images in Fiji and using the Reset function in the Adjust Brightness/Contrast menu, after average background intensity subtraction using the Math -> Subtract function. These average background intensity values were obtained by measuring a square area devoid of any cells.

For quantification of signal intensity in Fiji, a line was drawn along the longitudinal axis of bacterial cells and signal was measured using Plot Profile. Results of this function were saved as.csv files. For each strain, at least 50 bacterial cells were measured from raw images, using at least two different images for quantification. Average background values were subsequently subtracted from each results file using a.csv file containing the Plot Profile measurements of a linear area devoid of cells for each image used. Boxplots of fluorescence intensity and signal variance or pooled histograms were plotted using R [96]. The signal variance was obtained by calculating the variance of the log_2_ fold-change between each measured value and the average of all values for a given measured cell. This was done to normalize the data and obtain comparable variances without introducing a bias due to differences in overall signal intensity.

### Western Blot

Exponentially growing PAO1 strains expressing a GFP-tagged or Strep-tagged protein of interest were harvested in equal amounts and resuspended in a given volume of 1x Laemmli buffer before heating samples for 10 minutes at 95°C. Equal volumes of samples were run on a 4-12% Bis-Tris precast gel (Merck Millipore) at 130 V for at least 60 minutes and transferred onto a nitrocellulose blotting membrane using transfer buffer (3.03 g Tris base, 14.26 g glycine and 20% v/v ethanol per liter) and a Trans-Blot SD Semi Dry Transfer cell (0.15 A for 60 minutes). After blotting, the membrane was incubated overnight at 4°C in PBS-Tween 0.1% + 5% milk. Incubation with the primary antibody (StrepMAB-Classic, Iba; GFP Polyclonal antibodies, ChromoTek) diluted in PBS-Tween 0.1% + 5% milk was done for two hours. After three washing steps with PBS-Tween 0.1%, incubation with the secondary antibody (Anti-mouse or Anti-rabbit IgG-peroxidase antibodies, Sigma-Aldrich) diluted in PBS-Tween 0.1% + 5% milk was done for one hour, after which an additional three washes with PBS-Tween 0.1% were performed. A final wash with PBS was done before revelation and visualization of the signal using the SuperSignal West Pico Plus Chemiluminescent substrate (Thermofisher) and a ChemiDoc MP Imaging System (Bio-Rad).

### Pull-down identification of RNase E partners

Fresh cultures of PAO1 WT, PAO1 RNase E^1-529^-Strep, or PAO1 RNase E^1-1057^-Strep were inoculated to OD=0.05 from overnight cultures in 500 mL Erlenmeyer flasks. Cultures were grown until approx. OD_600nm_=0.8-0.9. 50 mL of each culture was harvested by centrifugation at 4500 *x g* for 10 minutes. Pellets were stored at -80°C until needed.

Each thawed pellet was then resuspended in 3,25 mL of lysis buffer (for 10 mL of buffer, prepared just before use: 20 mM Tris (pH 10), 250 mM NaCl, 10% v/v glycerol, 1 EDTA-free protease inhibitor tablet, 50 μg/mL lysozyme). Samples were incubated the samples on ice for about 30 minutes, transferred into 15-mL Falcon tubes and sonicated using a thin probe for 6x30s, with 15 seconds break in between. A pulse of 70% and a power output of 50% were used. After sonication, 32.5 μL of Triton X100 10% were added to each sample (~0.1% final) before incubation on a rotary wheel at 4°C for 8 minutes. The samples were transferred into 2 mL Eppendorf tubes and centrifuged for 1 hour at 10500 *x g* at 4°C. MagStrep Strep-Tactin XT beads (IBA, cat# 2-5090) were washed in wash buffer (20 mM Tris (pH 10), 250 mM NaCl, 10% v/v glycerol) according to the manufacturer’s instructions. One mix of beads was prepared for all the samples with a volume of beads suspension of 45 μL per sample. Final resuspension was done in 200 μL wash buffer per sample, plus an additional 10 μL. After centrifugation of the samples, the supernatant of each sample was regrouped into a 5 mL Eppendorf tube, and 200 μL of washed beads suspension were added per sample. Incubation on a rotary wheel was done for 2h15 at 4°C. The 5 mL tubes were then placed against a magnetic rack and all the supernatant was carefully removed. A first wash volume of 500 μL was added gently and agitated by horizontal rotations of the tubes. Tubes were placed back against the magnetic rack and the wash volume was removed. A second wash step was performed identically with a volume of 225 μL instead of 500 μL. A third wash step of 225 μL was also performed, and the resuspended beads were transferred into a clean 1.5 mL Eppendorf tube by gentle pipetting with a 1000 μL tip, before removing the wash volume. The beads were covered with 10 μL of sterile PBS and stored at 4°C before LC-MS/MS analysis at the Proteomics Core Facility (University of Geneva). For plots in Fig 3A and 3B, the log2 fold-change (log2FC) was calculated using the following formula: log2(average peptide count of protein 𝑥 identified in the RNase E^1-xxx^-Strep/ average peptide count of protein 𝑥 identified in the untagged sample).When an average count of 0 peptide was identified in the untagged sample, the log2FC value was instead calculated using the following formula: log2FC(average peptide count of protein 𝑥 identified in the RNase E^1-xxx^-Strep/1). For the RNase E^1-529^-Strep samples, when the average peptide count was equal to 0, the log2FC was assigned a value of 0 for plotting the data to avoid mathematical error and loss of data points on the graph.

### Bacterial adenylate cyclase two-hybrid assay

Cloned plasmids were introduced into chemically competent BTH101 cells by heat shock, and the cells were plated in LB agar supplemented with ampicillin and kanamycin. Isolated colonies were then streaked as a line of about 1-2 cm on freshly prepared Mac Conkey agar medium supplemented with ampicillin, kanamycin, 0.5 mM isopropyl β-d-thiogalactoside (IPTG), and maltose 1%. The MacConkey Petri dishes were then incubated at 30°C for 24h followed by an incubation at room temperature (~23°C) for 24h. The bacteria were imaged by scanning the plates, after which quantification of β-galactosidase activity was done using ONPG as described in [97].

### Protein purification

His_10_Smt3-tagged PNPase, His_10_Smt3-tagged RhlB, and His_10_Smt3-tagged ArcB proteins were purified from soluble bacterial lysates by nickel-agarose affinity chromatography as described previously in the supplementary information of Hausmann *et al.* 2021 [54]. The imidazole elution profiles were monitored by SDS-PAGE. The recombinant His_10_Smt3-tagged proteins were recovered predominantly in the 250 mM imidazole fraction. Protein concentration was determined using the Bio-Rad dye reagent, with BSA as the standard, and calculated by interpolation against the BSA standard curve. The His_10_-Smt3 tag remained uncleaved in the protein-protein interaction assays.

RNase E^1-1057^, RNase E^1-588^, RNase E^1-529^, RNase E^572-1057^, RNase E^572-1057_NDPRmut^, and RNase E^572-1057_AR1mut^ proteins, each carrying a His_10_Smt3 tag at the N-terminus and a Twin-Strep-Flag-tag at the C-terminus, were purified as described above. The 250 mM imidazole elution fractions were used directly in the Protein-Protein interaction assays. For the EMSA experiment, the RNase E proteins were further purified as follows: the 250 mM imidazole fraction were applied to a 1 ml of Strep-Tactin Sepharose (Iba) column that had been equilibrated with buffer C (50 mM Tris pH8, 200 mM NaCl, 10% glycerol). The column was washed with 5 ml of buffer C and then eluted stepwise with 1 ml aliquots of buffer C containing, 2.5 mM D-Desthiobiotin respectively. The elution profiles were monitored by SDS-PAGE.

### Protein-protein interaction assays

Anti-Flag M2 magnetic beads (Sigma M8823) were used for the binding assay (25 µL of beads suspension per reaction sample). The beads were washed once with 1 mL of buffer H (50 mM Tris-HCl, pH 8.0, 150 mM NaCl, 0.01% Triton X-100), before being resuspended in fresh buffer H and aliquoted into one Eppendorf tube per sample. Purified RNase E^1-529^-Flag, RNase E^1-588^-Flag, RNase E^572-1057^-Flag, RNase E^572-1057_NDPRmut^- Flag, or RNase E^572-1057_AR1mut^- Flag fusion proteins (20-25 µg each) were diluted in buffer H to a final volume of 100 µL and incubated with the magnetic beads for 1 hour at 4 °C on a rotating device. After the incubation, the beads were collected using a magnetic separator and washed three times with 0.25 ml of binding buffer H to remove any unbound protein.

For affinity chromatography, the anti-Flag magnetic beads (Sigma-Aldrich) with bound RNase E-Flag fusion proteins, or unbound beads, were incubated with either 5 µg of PNPase (Fig 4A) or 5 µg of RhlB (Fig 4B) diluted in buffer H (final volume 100 µL) for 1 hour at 4 °C on a rotating device. The control samples with no prey proteins were similarly incubated with 100 µL of buffer H. After incubation, the beads were collected using the magnet, and the supernatant (free fraction F) was withdrawn. The anti-Flag beads were then resuspended in 100 µl of binding buffer H and subjected to three rounds of washing of 100 µl each. Following the final wash, the proteins bound to the beads were eluted with 100 µl of 2X LDS sample buffer (Invitrogen) supplemented with 167 mM DTT. Aliquots (15 µl) of the input protein sample (PNPase or RhlB), the first supernatant fraction (free fraction F), and the bead-bound fraction were mixed with 5 µl of 4X LDS sample buffer (Invitrogen), heated at 80 °C for 5 minutes, and analysed by SDS-PAGE. Polypeptides were visualized using Coomassie Blue staining.

When performing the affinity chromatography with ArcB, the protocol described above was edited as follows. A volume of 50 µL beads was used per sample, and when using purified RNase E^1-1057^-Flag fusion protein, a volume of 450 µL of purified protein (in a buffer with 50 mM Tris pH 8, 500 mM NaCl, 10% glycerol and 250 mM imidazole) was incubated with the beads. After incubation, the washing steps were done using a volume of 800 µL of buffer H. The next steps were as described above,

except for the elution. To avoid co-migration of ArcB with the IgG from the beads (nearly identical molecular weight), specific elution with 100 µL of 250 ng/µL 3X FLAG Peptide (Sigma-Aldrich) was done for 30 min at 4°C on a rotary wheel followed by incubation at room temperature for 15 minutes.

### Growth assays on solid or liquid media

To assess the growth of the mutant strains in liquid medium, 200 μL of NYB broth was inoculated to OD_600nm_=0.05 in a 96-well plate, using an overnight culture washed twice with fresh medium. The wells on the edge of the plate were not used. The plate was incubated at 37°C using a Biotek Synergy H1 Hybrid Plate reader. OD_600nm_ values were measured every 30 minutes. Before each OD_600nm_ measurement, orbital shaking with 282 cpm (3 mm) was performed for 1 minute. Data was retrieved the following day and plotted after substraction of blank OD_600nm_ values using GraphPad Prism.

When growing strains carrying the pME6032 vector for complementation assays, NYB medium was supplemented with 50 μg/mL of Tetracycline and 70 μM of IPTG for induction. Cells were resuspended in supplemented NYB after washing and before inoculating the 96-well plate.

To assess the growth of the mutant strains on solid medium, square Petri dishes (12x12cm) were filled with 50 mL of warm NA. When testing growth at low vs high pH, either 75 μL of 37% fuming HCl or 400 μL of 5N NaOH was added per 50 mL of NA agar to achieve the desired conditions. A fresh culture (OD_600nm_= 0.4-0.8) of each strain was adjusted to OD_600nm_=1 by centrifugation and serially diluted in a 96-well plate using a multipipette. 5 μL of each dilution series was then spotted using a multipipette onto pre-dried NA plates (1 hour under a sterile flowhood). After the spots had dried, plates were incubated upside down at 37°C or 16°C.

### Galleria killing assays

*Galleria mellonella* larvae were used to assess in vivo virulence and/or survival of several RNase E mutant strains. Fresh cultures were adjusted to a suspension of OD_600nm_= 1 and serially diluted to 10^-6^ in Gibco Dulbecco’s Phosphate Buffered Saline (dPBS) (Thermo Fisher Scientific). 10 μL of the 10^-6^ dilution were spotted onto NYB agar to verify that the dilutions were similar for the different strains. 10 μL spots were then put onto a clean parafilm and injected using Omnican 50 sterile insulin syringe (Braun) into the second to last leg of the larvae, facing towards the head. As a control, sterile dPBS was injected similarly into larvae to assess background levels of death. For every 10 larvae injected, the time of injection was recorded, and they were placed inside a dark incubator at 37°C. The next day, deaths were recorded every hour. A larva was considered dead when it completely stopped responding to stimuli (touched on the legs or the head with a pipette tip). Time of death was calculated as the interval of time between recorded time of injection and death occurrence. Survival curves were plotted and analysed with GraphPad Prism (GraphPad Software, Prism10.2).

### RNA-seq sample collection

Independent triplicates cultures of PAO1 WT, PAO1 RNase E^1-588^, (*rne588*) and PAO1 RNase E^1-529^ (*rne529*) strains were grown in 50 mL Erlenmeyer flasks (10 mL NYB broth) until OD_600nm_= 1.0-1.2. 500 μL of each culture were immediately added into four separate 1.5 mL tubes, each containing 1 mL of RNAlater (Sigma-Aldrich). After harvesting of all three strains, the tubes were centrifuged for 30 minutes at 9600 x *g*. Supernatant was discarded and pellets were stored at -80°C until RNA extraction. The RNA extraction was performed using two tubes of pellets for each strain and replicate, with the Monarch Total RNA Miniprep kit. For lysis of the bacterial cells, the two pellets were resuspended in 250 μL of 1X Tris-EDTA (TE) buffer supplemented with 1 mg/mL of lysozyme and regrouped into one tube for each strain, incubated for 5 minutes at RT, and RNA was subsequently extracted following the manufacturer’s instructions. After RNA purification, an additional DNA removal treatment was done using TURBO DNase (Thermo Fisher) according to the manufacturer’s instructions. RNA was purified again using the RNA purification columns (dark blue) from the Monarch Total RNA Miniprep kit, and following the priming, washing and elution steps described by the manufacturer. The absence of residual gDNA contamination was verified by performing a PCR for 35 cycles using primer pairs targeting *rpoD* [54]. Biological triplicate samples for each strain were prepared. Quality check, ribosomal RNA depletion and RNA sequencing were conducted by Novogene.

### RNA sequencing analysis

Reads were mapped using Bowtie2 [98] to *Pseudomonas aeruginosa* PAO1 genome assembly ASM676v1 and counts were aggregated using summarize Overlaps in the package GenomicAlignments [99] in R [100] using the ASM676v1 gtf version 2.2 (NCBI accession: GCF_000006765.1). Differential expression was performed using DESeq2 [101] and transcripts were considered differentially expressed between two conditions if the absolute value of log2FC between conditions was greater than 1 (i.e. 2-fold up or down) with an adjusted p-value less than 0.05. To find functional enrichment among differentially expressed genes groups, the Kyoto Encyclopedia of Genes and Genomes (KEGG) was used for gene set definitions, downloaded using the package KEGGREST [102] in R. Enrichment was calculated using a hypergeometric test. All correlations presented are Pearson’s product moment correlation coefficient. Pairwise correlations of log2 Reads Per Kilobase per Million mapped reads (RPKMs) and hierarchical unsupervised clustering analysis of fold changes relative to WT demonstrate the similarity in the profile of the biological replicates for each strain. Analysis of 5’ read ends per loci was performed as described by de Araújo et al. [76] except that each dataset read number was down-sampled to 8 million. Raw counts of RNA seq are accessible in the NCBI GEO database with the accession number GSE287326.

### RT-qPCR

500 ng of RNA was mixed with an excess of dNTPs and random primers (Promega), incubated at 65°C for 5 minutes, then quickly transferred on ice before addition of ImpromII buffer, MgCl_2_, RNAsin, and ImpromII reverse transcriptase (concentrations according to manufacturer’s instruction, final volume 20 μL). Samples were incubated for 1h at 42°C followed by 15 minutes at 70°C to inactivate the enzyme. 180 μL of Ultrapure Distilled water (Invitrogen) were added to the cDNA samples. For the qPCR reactions, each WT, *rne529*, and *rne588* sample was run in three technical replicates and three biological replicates. 2,5 μL of cDNA were diluted in 17,5 μL of mix containing SensiFAST SYBR Hi-ROX mix and recommended dilutions of the primers of interest (S3 Table). Primer pairs q65-q66, q107-q109, and q110-q111 were used to amplify a 117-170 bp amplicon for *rpoD*, *rne* coding sequence (CDS), or *rne* 5’ untranslated region (UTR), respectively. The 96-well plate was sealed with Microseals B (Bio-Rad) and briefly centrifuged before running in a CFX96 Real-Time System C1000 Touch Thermocycler. The running conditions were as follows: 95°C for 2 min, 39 cycles of 95°C for 5s, 60°C for 20s, 72°C for 15s, melt curve 65°C to 95°C increment 0.5°C.

The 2⁻^ΔΔCt^ method was used to calculate the fold-change of *rne* CDS and *rne* 5’UTR relative to WT [103]. The *rpoD* gene was used as an internal control for the normalization of gene expression. The mean log₂ fold-change and standard error of the mean (SEM) were computed in R using tidyverse [104] and ggpubr [105] for data analysis and visualization. Unpaired two-tailed Student’s t-tests were applied to compare gene expression levels between conditions, with a significance threshold of p < 0.05.

## Supporting information

S1 Table List of strains used in this study. (DOCX)

S2 Table List of plasmids used and cloning details. (DOCX)

S3 Table List of primers used in this study. (DOCX)

S4 Table Sequence of mutated SLiMs. (DOCX)

S5 Table Excel file with data of the mass spectrometry analysis for identification of RNase E protein partners. (XLSX)

S6 Table Excel file with RNAseq data. Sheet A. Comparisons *rne529* vs *rne588* targets (_over WT),Sheet B. Differentially regulated sRNAs genes, Sheet C. KEGG enrichment: gene downregulated in the *rne529* strain vs WT, Sheet D. KEGG enrichment: gene upregulated in the *rne529* strain vs WT, Sheet E. KEGG enrichment: gene downregulated in the *rne588* strain vs WT,Sheet F. KEGG enrichment: gene upregulated in the *rne588* strain vs WT.
(XLSX)

S1 Fig Alignment of *E. coli* (Ec) and *P. aeruginosa* (Pa) RNase E homologs. Alignment of the Ec *rne* gene with the Pa homolog (PA2976) was done using the EBLOSUM62 matrix.
(DOCX)

S2 Fig Variability of RNase E SLiMs between P. aeruginosa and E. coli or other Pseudomonas species. **(A)** Comparison of *E. coli* (Ec) and *P. aeruginosa* (Pa) RNase E SLiMs based on the analysis performed in [29].
The precise amino acid positions were determined by manual sequence inspection. **(B)** Analysis of the frequency of RE [ED]R repeats within the *Pseudomonas* genus (see Materials and methods for details).
(EPS)

S3 Fig Logo plot displaying the consensus sequence of global alignment of RNase E homologs within the Pseudomonas group. 5000 RNase E homolog sequences were downloaded from the NCBI database, aligned using Clustal Omega and created using Weblogo [106].
Identified SLiMs are shown within boxes of different colours and all overlap with SLiMs previously identified in [29], though they are typically longer than previously described SLiMs. Position of the SLiMs on the RNase E protein of the *P. aeruginosa* PAO1 strain is indicated (see also Fig 1B).
(EPS)

S4 Fig RNase E CTD RNA binding: extended EMSA and extended microscopy analysis. **(A-B)** EMSA with purified RNase E C-terminal domain (residues 572-1057) having either the native sequence (WT) or systematic alanine mutation of arginine residues within the REER-repeats region (REERmut) (B), the AR4 SLiM (AR4mut) (A) or the AR1 SLiM (AR1mut) (A).
The *malEF* mRNA was used as a substrate at a concentration of 35 nM. Ratios of RNA:protein ranging from 1:0.3 to 1:10 (A) or 1:0.3 to 1:80 (B) were tested. **(C)** Quantification of average signal intensity in PAO1 strains shown in 2B Fig. I: RNase E-msfGFP, II: RNase E^1-588^-msfGFP, III: RNase E^1-529^-msfGFP, IV: RNase E^AR4mut^-msfGFP, V: RNase E^REERmut^-msfGFP, VI: RNase E^AR1mut^-msfGFP, VII: RNase E^AR1+AR4mut^-msfGFP, VIII: RNase E^AR1+AR4+REERmut^-msfGFP. Fluorescence intensity was measured along the longitudinal axis of the cells in 50-100 cells using Fiji. The intensity values were corrected by subtraction of background noise values. Quantification of the variance of pixel intensity normalised by the average cell intensity was done using R. Statistical significance was assessed by performing t-tests using R. p-values for comparisons tested are indicated as follows: *:<0.05; **:<0.01; ***:<0.001; ****:<0.0001, n.s.: not significant (95% confidence interval), N.T.: not tested. **(D)** Western Blot showing expression levels of chromosomal Strep-tagged RNase E variants. L: protein ladder, 1: WT RNase E, 2: RNase E^1-588^, 3: RNase E^1-529^, 4: RNase E^AR1mut^, 5: RNase E^AR1+AR4+REERmut^. As a loading control, the expression levels of the major outer membrane protein OprF were probed using anti-OprF antibodies. **(E)** Representative images of live PAO1 strains expressing truncated variants of the RNase E-msfGFP fusion as described above each image. The scale is 2 μm. **(F)** Pooled histograms showing the distribution of the measured signal intensity corrected by the mean signal intensity in the corresponding cell. Representative images of each RNase E-msfGFP variant or exposure conditions are shown in Fig 2B and 2D.
(EPS)

S5 Fig Western Blot analysis of msfGFP-tagged RNase E truncated or mutated variants. Anti-GFP (for probing RNase E proteins) and anti-OprF (loading control) Western Blot were performed from crude lysates of strains expressing one of the following msfGFP-tagged RNase E variants from the native locus and promoter, replacing the WT copy.
L: Protein ladder; 1: RNase E-msfGFP; 2: RNase E^AR4mut^-msfGFP; 3: RNase E^AR1mut^-msfGFP; 4: RNase E^REERmut^-msfGFP; 5: RNase E^AR1+AR4mut^-msfGFP; 6: RNase E^AR1+AR4+REERmut^-msfGFP; 7: RNase E^1-588^-msfGFP; 8: RNase E^1-529^-msfGFP; 9: RNase E-msfGFP no Rifampicin (DMSO only); 10: RNase E-msfGFP + Rifampicin 100 μg/mL (20 mg/mL stock in DMSO).
(EPS)

S6 Fig Purified proteins, pull-down eluates analysis, and extended direct binding assays. **(A)** SDS-PAGE gel showing the single (His_10_-Smt3) Flag-tagged purified proteins used as input for the direct binding assays (RNase E^1-529^ and RNase E^1-588^) and the double purified proteins used in the EMSA experiments (RNase E^572-1057^ WT and variants).
**(B)** Silver staining of pull-down eluates obtained using RNase E^1-1057^-Strep or RNase E^1-529^-Strep as a bait protein. The negative control was done using cellular lysates from the WT strain (untagged). Direct resuspension of the MagStrep Strep-Tactin XT magnetic beads in 1x Laemmli buffer was done. kDa: kiloDaltons. The ProteoSilver Silver Stain kit (Sigma) was used according to the manufacturer’s instructions. **(C)** Direct protein-protein binding assay between PNPase and RNase E^1-588^. I: input PNPase fraction, F: free PNPase fraction, B: bound PNPase fraction. **(D)** Direct protein-protein binding assay between ArcB and RNase E^1-1057^ or RNase E^1-588^ in presence or absence of 100 ng/µl of total *P. aeruginosa* RNA. I: input ArcB fraction, F: free ArcB fraction, E: eluted fraction. The binding assay was performed twice, and a representative gel is shown. For **(C-D)**, purified His_10_-Smt3-RNase E^1-1057^-Strep-FLAG or His_10_-Smt3-RNase E^1-588^-Strep-FLAG proteins were used as baits, immobilized to anti-flag magnetic beads and incubated with purified His_10_-Smt3-PNPase, His_10_-Smt3-ArcB or buffer alone as indicated to assess direct protein binding.
(EPS)

S7 Fig Bacterial two hybrid (BTH) results. Visualisation of the bacterial two-hybrid E. coli BTH101 patches used for quantification of β-galactosidase activity (Fig 3C-3F). Isolated colonies were patched onto McConkey agar, allowing visualisation of a red pigmentation if the bacteria are able to metabolise maltose and lactose due to restored cAMP production.
**(A)** *E. coli* BTH101 co-expressing T18-RNase E with one of seven T25-fused putative protein partners, as indicated below each picture. **(B-D)** *E. coli* BTH101 co-expressing T25-PNPase **(B)**, T25-RhlB **(C)**, or T25-ArcB **(D)** with either T18-RNase E or one of eight to ten truncated T18-RNase E fragments, as indicated below each picture.
(EPS)

S8 Fig Growth of chromosomally tagged strains and subcellular localisation of RNase E and/or ArcB. **(A)** Growth curves of PAO1 strains expressing chromosomal msfGFP-tagged or mCherry-tagged versions of RNase E (*rne::msfGFP*/ *rne::mCherry*), PNPase (*pnp::msfGFP*), or RhlB (*rhl::msfGFP*).
Bacteria were cultivated in NYB medium at 37°C in a 96-well plate (see Materials and methods). **(B-C)** Representative images of a strain expressing RNase E^NDPRmut^-msfGFP **(B)**, highlighting the formation of foci similar to those observed for RNase E-msfGFP (shown in Fig 2B), or a strain co-expressing ArcB-msfGFP and RNase E-mCherry **(C)**, showing a completely smooth subcellular localisation for ArcB-msfGFP rather than a foci localisation which is expected for a core RNA degradosome component. Scale is 2 μm. The images were corrected for background noise as described in Materials and methods.
(EPS)

S9 Fig Complementation assay. Expression of RNase E, RNase E-msfGFP or RNase E-mCherry from the IPTG-inducible pME6032 vector can complement the growth delay of rne529 or rneAR1+AR4+REERmut chromosomal mutant strains at 37°C. For reference, the WT strain was also transformed with the pME6032 vectors. For growth details, see Materials and Methods.
(EPS)

S10 Fig RNA sequencing: quality control and assessment of similarity between replicates and comparison of strains. **(A)** Pairwise Pearson correlation of log10 RPKMs across all samples. **(B)** Unsupervised hierarchical clustering of log10 RPKMs using a Euclidiean distance measure.
(EPS)

S11 Fig Histogram of the ratio of coverage in each sample compared to each WT sample across all genomic positions. Each replicate of every strain is compared, pairwise with each WT replicate. For each genomic position, a ratio is calculated: the coverage of 5’ends of reads in the first sample is divided by the total coverage across this sample and WT replicate (all samples are down-sampled to 8 million reads). A histogram of the frequencies of these ratios is plotted. The primary focus is whether we see an enrichment of ratios of 1 in the deletion, indicating an increase in positions solely with 5’end coverage in the deletion, suggesting potential cleavage points not present in WT.
(EPS)

## References

[1] Hui MP, Foley PL, Belasco JG. Messenger RNA degradation in bacterial cells. Annu Rev Genet. 2014;48:537–59. doi: 10.1146/annurev-genet-120213-092340 25292357 PMC4431577

[2] Aït-Bara S, Carpousis AJ. RNA degradosomes in bacteria and chloroplasts: classification, distribution and evolution of RNase E homologs. Mol Microbiol. 2015;97(6):1021–135. doi: 10.1111/mmi.13095 26096689

[3] Carpousis AJ. The RNA degradosome of Escherichia coli: an mRNA-degrading machine assembled on RNase E. Annu Rev Microbiol. 2007;61:71–87. doi: 10.1146/annurev.micro.61.080706.093440 17447862

[4] Tejada-Arranz A, de Crécy-Lagard V, de Reuse H. Bacterial RNA Degradosomes: Molecular Machines under Tight Control. Trends Biochem Sci. 2020;45(1):42–57. doi: 10.1016/j.tibs.2019.10.002 31679841 PMC6958999

[5] Dressaire C, Picard F, Redon E, Loubière P, Queinnec I, Girbal L, et al. Role of mRNA stability during bacterial adaptation. PLoS One. 2013;8(3):e59059. doi: 10.1371/journal.pone.0059059 23516597 PMC3596320

[6] Broglia L, Le Rhun A, Charpentier E. Methodologies for bacterial ribonuclease characterization using RNA-seq. FEMS Microbiol Rev. 2023;47(5):fuad049. doi: 10.1093/femsre/fuad049 37656885 PMC10503654

[7] Ono M, Kuwano M. A conditional lethal mutation in an *Escherichia coli* strain with a longer chemical lifetime of messenger RNA. J Mol Biol. 1979;129(3):343–57. doi: 10.1016/0022-2836(79)90500-x 110942

[8] Apirion D. Isolation, genetic mapping and some characterization of a mutation in *Escherichia coli* that affects the processing of ribonuleic acid. Genetics. 1978;90(4):659–71. doi: 10.1093/genetics/90.4.659 369943 PMC1213911

[9] Jain C, Deana A, Belasco JG. Consequences of RNase E scarcity in *Escherichia coli*. Mol Microbiol. 2002;43(4):1053–64. doi: 10.1046/j.1365-2958.2002.02808.x 11929550

[10] Caruthers JM, Feng Y, McKay DB, Cohen SN. Retention of core catalytic functions by a conserved minimal ribonuclease E peptide that lacks the domain required for tetramer formation. J Biol Chem. 2006;281(37):27046–51. doi: 10.1074/jbc.M602467200 16854990

[11] Callaghan AJ, Aurikko JP, Ilag LL, Günter Grossmann J, Chandran V, Kühnel K, et al. Studies of the RNA degradosome-organizing domain of the *Escherichia coli* ribonuclease RNase E. J Mol Biol. 2004;340(5):965–79. doi: 10.1016/j.jmb.2004.05.046 15236960

[12] Carpousis AJ, Van Houwe G, Ehretsmann C, Krisch HM. Copurification of E. coli RNAase E and PNPase: evidence for a specific association between two enzymes important in RNA processing and degradation. Cell. 1994;76(5):889–900. doi: 10.1016/0092-8674(94)90363-8 7510217

[13] Py B, Higgins CF, Krisch HM, Carpousis AJ. A DEAD-box RNA helicase in the *Escherichia coli* RNA degradosome. Nature. 1996;381(6578):169–72. doi: 10.1038/381169a0 8610017

[14] Chandran V, Luisi BF. Recognition of enolase in the *Escherichia coli* RNA degradosome. J Mol Biol. 2006;358(1):8–15. doi: 10.1016/j.jmb.2006.02.012 16516921

[15] Worrall JAR, Górna M, Crump NT, Phillips LG, Tuck AC, Price AJ, et al. Reconstitution and analysis of the multienzyme *Escherichia coli* RNA degradosome. J Mol Biol. 2008;382(4):870–83. doi: 10.1016/j.jmb.2008.07.059 18691600 PMC7611026

[16] Bandyra KJ, Bouvier M, Carpousis AJ, Luisi BF. The social fabric of the RNA degradosome. Biochim Biophys Acta. 2013;1829(6–7):514–22. doi: 10.1016/j.bbagrm.2013.02.011 23459248 PMC3991390

[17] Tsai Y-C, Du D, Domínguez-Malfavón L, Dimastrogiovanni D, Cross J, Callaghan AJ, et al. Recognition of the 70S ribosome and polysome by the RNA degradosome in *Escherichia coli*. Nucleic Acids Res. 2012;40(20):10417–31. doi: 10.1093/nar/gks739 22923520 PMC3488216

[18] Leroy A, Vanzo NF, Sousa S, Dreyfus M, Carpousis AJ. Function in *Escherichia coli* of the non-catalytic part of RNase E: role in the degradation of ribosome-free mRNA. Mol Microbiol. 2002;45(5):1231–43. doi: 10.1046/j.1365-2958.2002.03104.x 12207692

[19] Dendooven T, Paris G, Shkumatov AV, Islam MS, Burt A, Kubańska MA, et al. Multi-scale ensemble properties of the *Escherichia coli* RNA degradosome. Mol Microbiol. 2022;117(1):102–20. doi: 10.1111/mmi.14800 34415624 PMC7613265

[20] Sinha D, De Lay NR. Target recognition by RNase E RNA-binding domain AR2 drives sRNA decay in the absence of PNPase. Proc Natl Acad Sci U S A. 2022;119(48):e2208022119. doi: 10.1073/pnas.2208022119 36409892 PMC9860253

[21] Lee K, Zhan X, Gao J, Qiu J, Feng Y, Meganathan R, et al. RraA. a protein inhibitor of RNase E activity that globally modulates RNA abundance in *E. coli*. Cell. 2003;114(5):623–34. doi: 10.1016/s0092-8674(03)00646-9 13678585

[22] Górna MW, Pietras Z, Tsai Y-C, Callaghan AJ, Hernández H, Robinson CV, et al. The regulatory protein RraA modulates RNA-binding and helicase activities of the *E. coli* RNA degradosome. RNA. 2010;16(3):553–62. doi: 10.1261/rna.1858010 20106955 PMC2822920

[23] Gao J, Lee K, Zhao M, Qiu J, Zhan X, Saxena A, et al. Differential modulation of E. coli mRNA abundance by inhibitory proteins that alter the composition of the degradosome. Mol Microbiol. 2006;61(2):394–406. doi: 10.1111/j.1365-2958.2006.05246.x 16771842

[24] Prud’homme-Généreux A, Beran RK, Iost I, Ramey CS, Mackie GA, Simons RW. Physical and functional interactions among RNase E, polynucleotide phosphorylase and the cold-shock protein, CsdA: evidence for a “cold shock degradosome”. Mol Microbiol. 2004;54(5):1409–21. doi: 10.1111/j.1365-2958.2004.04360.x 15554978

[25] Blum E, Py B, Carpousis AJ, Higgins CF. Polyphosphate kinase is a component of the Escherichia coli RNA degradosome. Mol Microbiol. 1997;26(2):387–98. doi: 10.1046/j.1365-2958.1997.5901947.x 9383162

[26] Kannaiah S, Goldberger O, Alam N, Barnabas G, Pozniak Y, Nussbaum-Shochat A, et al. MinD-RNase E interplay controls localization of polar mRNAs in *E. coli*. EMBO J. 2024;43(4):637–62. doi: 10.1038/s44318-023-00026-9 38243117 PMC10897333

[27] Taghbalout A, Rothfield L. RNaseE and the other constituents of the RNA degradosome are components of the bacterial cytoskeleton. Proc Natl Acad Sci U S A. 2007;104(5):1667–72. doi: 10.1073/pnas.0610491104 17242352 PMC1785250

[28] Ikeda Y, Yagi M, Morita T, Aiba H. Hfq binding at RhlB-recognition region of RNase E is crucial for the rapid degradation of target mRNAs mediated by sRNAs in *Escherichia coli*. Mol Microbiol. 2011;79(2):419–32. doi: 10.1111/j.1365-2958.2010.07454.x 21219461

[29] Aït-Bara S, Carpousis AJ, Quentin Y. RNase E in the γ-Proteobacteria: conservation of intrinsically disordered noncatalytic region and molecular evolution of microdomains. Mol Genet Genomics. 2015;290(3):847–62. doi: 10.1007/s00438-014-0959-5 25432321 PMC4435900

[30] Goldblum K, Apririon D. Inactivation of the ribonucleic acid-processing enzyme ribonuclease E blocks cell division. J Bacteriol. 1981;146(1):128–32. doi: 10.1128/jb.146.1.128-132.1981 6163761 PMC217061

[31] Bernstein JA, Lin P-H, Cohen SN, Lin-Chao S. Global analysis of *Escherichia coli* RNA degradosome function using DNA microarrays. Proc Natl Acad Sci U S A. 2004;101(9):2758–63. doi: 10.1073/pnas.0308747101 14981237 PMC365694

[32] Kido M, Yamanaka K, Mitani T, Niki H, Ogura T, Hiraga S. RNase E polypeptides lacking a carboxyl-terminal half suppress a mukB mutation in *Escherichia coli*. J Bacteriol. 1996;178(13):3917–25. doi: 10.1128/jb.178.13.3917-3925.1996 8682798 PMC232654

[33] Lopez PJ, Marchand I, Joyce SA, Dreyfus M. The C-terminal half of RNase E, which organizes the *Escherichia coli* degradosome, participates in mRNA degradation but not rRNA processing in vivo. Mol Microbiol. 1999;33(1):188–99. doi: 10.1046/j.1365-2958.1999.01465.x 10411735

[34] Hardwick SW, Chan VSY, Broadhurst RW, Luisi BF. An RNA degradosome assembly in *Caulobacter crescentus*. Nucleic Acids Res. 2011;39(4):1449–59. doi: 10.1093/nar/gkq928 20952404 PMC3045602

[35] Voss JE, Luisi BF, Hardwick SW. Molecular recognition of RhlB and RNase D in the *Caulobacter crescentus* RNA degradosome. Nucleic Acids Res. 2014;42(21):13294–305. doi: 10.1093/nar/gku1134 25389270 PMC4245959

[36] Zhang J-Y, Deng X-M, Li F-P, Wang L, Huang Q-Y, Zhang C-C, et al. RNase E forms a complex with polynucleotide phosphorylase in cyanobacteria via a cyanobacterial-specific nonapeptide in the noncatalytic region. RNA. 2014;20(4):568–79. doi: 10.1261/rna.043513.113 24563514 PMC3964918

[37] Yan H, Qin X, Wang L, Chen W. Both Enolase and the DEAD-Box RNA Helicase CrhB Can Form Complexes with RNase E in *Anabaena sp*. Strain PCC 7120. Appl Environ Microbiol. 2020;86(13):e00425-20. doi: 10.1128/AEM.00425-20 32303553 PMC7301867

[38] Zhou C, Zhang J, Hu X, Li C, Wang L, Huang Q, et al. RNase II binds to RNase E and modulates its endoribonucleolytic activity in the cyanobacterium *Anabaena* PCC 7120. Nucleic Acids Res. 2020;48(7):3922–34. doi: 10.1093/nar/gkaa092 32055835 PMC7144899

[39] Purusharth RI, Klein F, Sulthana S, Jäger S, Jagannadham MV, Evguenieva-Hackenberg E, et al. Exoribonuclease R interacts with endoribonuclease E and an RNA helicase in the psychrotrophic bacterium Pseudomonas syringae Lz4W*. J Biol Chem. 2005;280(15):14572–8. doi: 10.1074/jbc.M413507200 15705581

[40] Jäger S, Fuhrmann O, Heck C, Hebermehl M, Schiltz E, Rauhut R, et al. An mRNA degrading complex in *Rhodobacter capsulatus*. Nucleic Acids Res. 2001;29(22):4581–8. doi: 10.1093/nar/29.22.4581 11713307 PMC92556

[41] Aït-Bara S, Carpousis AJ. Characterization of the RNA degradosome of *Pseudoalteromonas haloplanktis*: conservation of the RNase E-RhlB interaction in the gammaproteobacteria. J Bacteriol. 2010;192(20):5413–23. doi: 10.1128/JB.00592-10 20729366 PMC2950506

[42] Hardwick SW, Gubbey T, Hug I, Jenal U, Luisi BF. Crystal structure of *Caulobacter crescentus* polynucleotide phosphorylase reveals a mechanism of RNA substrate channelling and RNA degradosome assembly. Open Biol. 2012;2(4):120028. doi: 10.1098/rsob.120028 22724061 PMC3376730

[43] Nurmohamed S, Vaidialingam B, Callaghan AJ, Luisi BF. Crystal structure of Escherichia coli polynucleotide phosphorylase core bound to RNase E, RNA and manganese: implications for catalytic mechanism and RNA degradosome assembly. J Mol Biol. 2009;389(1):17–33. doi: 10.1016/j.jmb.2009.03.051 19327365 PMC2723993

[44] Green SK, Schroth MN, Cho JJ, Kominos SK, Vitanza-jack VB. Agricultural plants and soil as a reservoir for *Pseudomonas aeruginosa*. Appl Microbiol. 1974;28(6):987–91. doi: 10.1128/am.28.6.987-991.1974 4217591 PMC186868

[45] Hardalo C, Edberg SC. *Pseudomonas aeruginosa*: assessment of risk from drinking water. Crit Rev Microbiol. 1997;23(1):47–75. doi: 10.3109/10408419709115130 9097014

[46] Pendleton JN, Gorman SP, Gilmore BF. Clinical relevance of the ESKAPE pathogens. Expert Rev Anti Infect Ther. 2013;11(3):297–308. doi: 10.1586/eri.13.12 23458769

[47] https://www.who.int/news/item/27-02-2017-who-publishes-list-of-bacteria-for-which-new-antibiotics-are-urgently-needed

[48] Garcia-Clemente M, de la Rosa D, Máiz L, Girón R, Blanco M, Olveira C, et al. Impact of Pseudomonas aeruginosa Infection on Patients with Chronic Inflammatory Airway Diseases. J Clin Med. 2020;9(12):3800. doi: 10.3390/jcm9123800 33255354 PMC7760986

[49] Urwin L, Okurowska K, Crowther G, Roy S, Garg P, Karunakaran E, et al. Corneal Infection Models: Tools to Investigate the Role of Biofilms in Bacterial Keratitis. Cells. 2020;9(11):2450. doi: 10.3390/cells9112450 33182687 PMC7696224

[50] Costerton JW, Stewart PS, Greenberg EP. Bacterial biofilms: a common cause of persistent infections. Science (NY). 1999;284(5418):1318–22. doi: 10.1126/science.284.5418.1318 10334980

[51] Lu Q, Eggimann P, Luyt C-E, Wolff M, Tamm M, François B, et al. *Pseudomonas aeruginosa* serotypes in nosocomial pneumonia: prevalence and clinical outcomes. Crit Care (London, England). 2014;18(1):R17. doi: 10.1186/cc13697 24428878 PMC4057348

[52] Hancock RE. Resistance mechanisms in *Pseudomonas aeruginosa* and other nonfermentative gram-negative bacteria. Clin Infect Dis. 1998;27 Suppl 1:S93-9. doi: 10.1086/514909 9710677

[53] Van den Bossche A, Hardwick SW, Ceyssens P-J, Hendrix H, Voet M, Dendooven T, et al. Structural elucidation of a novel mechanism for the bacteriophage-based inhibition of the RNA degradosome. Elife. 2016;5:e16413. doi: 10.7554/eLife.16413 27447594 PMC4980113

[54] Hausmann S, Gonzalez D, Geiser J, Valentini M. The DEAD-box RNA helicase RhlE2 is a global regulator of *Pseudomonas aeruginosa* lifestyle and pathogenesis. Nucleic Acids Res. 2021;49(12):6925–40. doi: 10.1093/nar/gkab503 34151378 PMC8266600

[55] Hausmann S, Geiser J, Allen GE, Geslain SAM, Valentini M. Intrinsically disordered regions regulate RhlE RNA helicase functions in bacteria. Nucleic Acids Res. 2024;52(13):7809–24. doi: 10.1093/nar/gkae511 38874491 PMC11260450

[56] Sonnleitner E, Romeo A, Bläsi U. Small regulatory RNAs in *Pseudomonas aeruginosa*. RNA Biol. 2012;9(4):364–71. doi: 10.4161/rna.19231 22336763

[57] Valentini M, Gonzalez D, Mavridou DA, Filloux A. Lifestyle transitions and adaptive pathogenesis of *Pseudomonas aeruginosa*. Curr Opin Microbiol. 2018;41 15–20. doi: 10.1016/j.mib.2017.11.006 29166621

[58] Erdős G, Pajkos M, Dosztányi Z. IUPred3: prediction of protein disorder enhanced with unambiguous experimental annotation and visualization of evolutionary conservation. Nucleic Acids Res. 2021;49(W1):W297–303. doi: 10.1093/nar/gkab408 34048569 PMC8262696

[59] Xue B, Dunbrack RL, Williams RW, Dunker AK, Uversky VN. PONDR-FIT: a meta-predictor of intrinsically disordered amino acids. Biochim Biophys Acta. 2010;1804(4):996–1010. doi: 10.1016/j.bbapap.2010.01.011 20100603 PMC2882806

[60] Nishio S, Itoh T. Arginine-rich RNA binding domain and protein scaffold domain of RNase E are important for degradation of RNAI but not for that of the Rep mRNA of the ColE2 plasmid. Plasmid. 2009;62(2):83–7. doi: 10.1016/j.plasmid.2009.04.002 19426759

[61] McLaren RS, Newbury SF, Dance GS, Causton HC, Higgins CF. mRNA degradation by processive 3’-5’ exoribonucleases in vitro and the implications for prokaryotic mRNA decay in vivo. J Mol Biol. 1991;221(1):81–95. doi: 10.1016/0022-2836(91)80206-a 1920421

[62] Hamouche L, Poljak L, Carpousis AJ. Polyribosome-Dependent Clustering of Membrane-Anchored RNA Degradosomes To Form Sites of mRNA Degradation in *Escherichia coli*. mBio. 2021;12(5):e0193221. doi: 10.1128/mBio.01932-21 34488454 PMC8546579

[63] Al-Husini N, Tomares DT, Bitar O, Childers WS, Schrader JM. α-Proteobacterial RNA Degradosomes Assemble Liquid-Liquid Phase-Separated RNP Bodies. Mol Cell. 2018;71(6):1027-1039.e14. doi: 10.1016/j.molcel.2018.08.003 30197298 PMC6151146

[64] Khemici V, Poljak L, Luisi BF, Carpousis AJ. The RNase E of *Escherichia coli* is a membrane-binding protein. Mol Microbiol. 2008;70(4):799–813. doi: 10.1111/j.1365-2958.2008.06454.x 18976283 PMC7610891

[65] Schuck A, Diwa A, Belasco JG. RNase E autoregulates its synthesis in Escherichia coli by binding directly to a stem-loop in the rne 5’ untranslated region. Mol Microbiol. 2009;72(2):470–8. doi: 10.1111/j.1365-2958.2009.06662.x 19320830 PMC2857391

[66] Liu S-J, Lin G-M, Yuan Y-Q, Chen W, Zhang J-Y, Zhang C-C. A conserved protein inhibitor brings under check the activity of RNase E in cyanobacteria. Nucleic Acids Res. 2024;52(1):404–19. doi: 10.1093/nar/gkad1094 38000383 PMC10783494

[67] Karimova G, Ullmann A, Ladant D. A bacterial two-hybrid system that exploits a cAMP signaling cascade in Escherichia coli. In: Thorner J, Emr SD, Abelson JN, editors. Methods in Enzymology. 328: Academic Press; 2000. p. 59-73. 10.1016/s0076-6879(00)28390-0 11075338

[68] Bolte S, Cordelières FP. A guided tour into subcellular colocalization analysis in light microscopy. J Microsc. 2006;224(Pt 3):213–32. doi: 10.1111/j.1365-2818.2006.01706.x 17210054

[69] Jander G, Rahme LG, Ausubel FM. Positive correlation between virulence of *Pseudomonas aeruginosa* mutants in mice and insects. J Bacteriol. 2000;182(13):3843–5. doi: 10.1128/JB.182.13.3843-3845.2000 10851003 PMC94559

[70] Lin J, Cheng J, Wang Y, Shen X. The Pseudomonas Quinolone Signal (PQS): Not Just for Quorum Sensing Anymore. Front Cell Infect Microbiol. 2018;8:230. doi: 10.3389/fcimb.2018.00230 30023354 PMC6039570

[71] Gonçalves T, Vasconcelos U. Colour Me Blue: The History and the Biotechnological Potential of Pyocyanin. Molecules. 2021;26(4):927. doi: 10.3390/molecules26040927 33578646 PMC7916356

[72] Chou HT, Li J-Y, Lu C-D. Functional characterization of the agtABCD and agtSR operons for 4-aminobutyrate and 5-aminovalerate uptake and regulation in *Pseudomonas aeruginosa* PAO1. Curr Microbiol. 2014;68(1):59–63. doi: 10.1007/s00284-013-0446-y 23982201

[73] Hauser AR. The type III secretion system of *Pseudomonas aeruginosa*: infection by injection. Nat Rev Microbiol. 2009;7(9):654–65. doi: 10.1038/nrmicro2199 19680249 PMC2766515

[74] Nandana V, Al-Husini N, Vaishnav A, Dilrangi KH, Schrader JM. Caulobacter crescentus RNase E condensation contributes to autoregulation and fitness. Mol Biol Cell. 2024;35(8):ar104. doi: 10.1091/mbc.E23-12-0493 38865176 PMC11321048

[75] Wurtzel O, Yoder-Himes DR, Han K, Dandekar AA, Edelheit S, Greenberg EP, et al. The single-nucleotide resolution transcriptome of *Pseudomonas aeruginosa* grown in body temperature. PLoS Pathog. 2012;8(9):e1002945. doi: 10.1371/journal.ppat.1002945 23028334 PMC3460626

[76] de Araújo HL, Picinato BA, Lorenzetti APR, Muthunayake NS, Rathnayaka-Mudiyanselage IW, Dos Santos NM, et al. The DEAD-box RNA helicase RhlB is required for efficient RNA processing at low temperature in Caulobacter. Microbiol Spectr. 2023;11(6):e0193423. doi: 10.1128/spectrum.01934-23 37850787 PMC10715135

[77] Vaillancourt M, Galdino ACM, Limsuwannarot SP, Celedonio D, Dimitrova E, Broerman M, et al. A compensatory RNase E variation increases Iron Piracy and Virulence in multidrug-resistant *Pseudomonas aeruginosa* during Macrophage infection. PLoS Pathog. 2023;19(4):e1010942. doi: 10.1371/journal.ppat.1010942 37027441 PMC10115287

[78] Khemici V, Toesca I, Poljak L, Vanzo NF, Carpousis AJ. The RNase E of *Escherichia coli* has at least two binding sites for DEAD-box RNA helicases: functional replacement of RhlB by RhlE. Mol Microbiol. 2004;54(5):1422–30. doi: 10.1111/j.1365-2958.2004.04361.x 15554979

[79] Worrall JAR, Howe FS, McKay AR, Robinson CV, Luisi BF. Allosteric activation of the ATPase activity of the *Escherichia coli* RhlB RNA helicase. J Biol Chem. 2008;283(9):5567–76. doi: 10.1074/jbc.M708620200 18165229 PMC7611231

[80] Lüthi E, Mercenier A, Haas D. The arcABC operon required for fermentative growth of Pseudomonas aeruginosa on arginine: Tn5-751-assisted cloning and localization of structural genes. J Gen Microbiol. 1986;132(10):2667–75. doi: 10.1099/00221287-132-10-2667 3040889

[81] Gamper M, Ganter B, Polito MR, Haas D. RNA processing modulates the expression of the arcDABC operon in *Pseudomonas aeruginosa*. J Mol Biol. 1992;226(4):943–57. doi: 10.1016/0022-2836(92)91044-p 1325563

[82] Chen R, Weng Y, Zhu F, Jin Y, Liu C, Pan X, et al. Polynucleotide Phosphorylase Regulates Multiple Virulence Factors and the Stabilities of Small RNAs RsmY/Z in Pseudomonas aeruginosa. Front Microbiol. 2016;7:247. doi: 10.3389/fmicb.2016.00247 26973625 PMC4773659

[83] Li K, Xu C, Jin Y, Sun Z, Liu C, Shi J, et al. SuhB is a regulator of multiple virulence genes and essential for pathogenesis of *Pseudomonas aeruginosa*. mBio. 2013;4(6):e00419-13. doi: 10.1128/mBio.00419-13 24169572 PMC3809559

[84] Fan Z, Chen H, Li M, Pan X, Fu W, Ren H, et al. *Pseudomonas aeruginosa* Polynucleotide Phosphorylase Contributes to Ciprofloxacin Resistance by Regulating PrtR. Front Microbiol. 2019;10 1762. doi: 10.3389/fmicb.2019.01762 31417536 PMC6682600

[85] Fan Z, Pan X, Wang D, Chen R, Fu T, Yang B, et al. *Pseudomonas aeruginosa* Polynucleotide Phosphorylase Controls Tolerance to Aminoglycoside Antibiotics by Regulating the MexXY Multidrug Efflux Pump. Antimicrob Agents Chemother. 2021;65(2):e01846-20. doi: 10.1128/AAC.01846-20 33257447 PMC7849007

[86] Rietsch A, Vallet-Gely I, Dove SL, Mekalanos JJ. ExsE, a secreted regulator of type III secretion genes in Pseudomonas aeruginosa. Proc Natl Acad Sci U S A. 2005;102(22):8006–11. doi: 10.1073/pnas.0503005102 15911752 PMC1142391

[87] Laville J, Voisard C, Keel C, Maurhofer M, Défago G, Haas D. Global control in *Pseudomonas fluorescens* mediating antibiotic synthesis and suppression of black root rot of tobacco. Proc Natl Acad Sci U S A. 1992;89(5):1562–6. doi: 10.1073/pnas.89.5.1562 1311842 PMC48492

[88] Ye RW, Haas D, Ka JO, Krishnapillai V, Zimmermann A, Baird C, et al. Anaerobic activation of the entire denitrification pathway in *Pseudomonas aeruginosa* requires Anr, an analog of Fnr. J Bacteriol. 1995;177(12):3606–9. doi: 10.1128/jb.177.12.3606-3609.1995 7768875 PMC177071

[89] Altschul SF, Gish W, Miller W, Myers EW, Lipman DJ. Basic local alignment search tool. J Mol Biol. 1990;215(3):403–10. doi: 10.1016/S0022-2836(05)80360-2 2231712

[90] Pagès HAP, Gentleman R, DebRoy S. Biostrings: Efficient manipulation of biological strings. 2024.

[91] Grant BJ, Rodrigues APC, ElSawy KM, McCammon JA, Caves LSD. Bio3d: an R package for the comparative analysis of protein structures. Bioinformatics. 2006;22(21):2695–6. doi: 10.1093/bioinformatics/btl461 16940322

[92] Edgar RC. MUSCLE: multiple sequence alignment with high accuracy and high throughput. Nucleic Acids Res. 2004;32(5):1792–7. doi: 10.1093/nar/gkh340 15034147 PMC390337

[93] Mackie GA, Coburn GA, Miao X, Briant DJ, Prud’homme-Généreux A, Stickney LM, et al. Preparation of the *Escherichia coli* RNase E protein and reconstitution of the RNA degradosome. Methods Enzymol. 2008;447:199–213. doi: 10.1016/S0076-6879(08)02211-8 19161845

[94] Ream JA, Lewis LK, Lewis KA. Rapid agarose gel electrophoretic mobility shift assay for quantitating protein: RNA interactions. Anal Biochem. 2016;511:36–41. doi: 10.1016/j.ab.2016.07.027 27495142 PMC5002362

[95] Schindelin J, Arganda-Carreras I, Frise E, Kaynig V, Longair M, Pietzsch T, et al. Fiji: an open-source platform for biological-image analysis. Nat Methods. 2012;9(7):676–82. doi: 10.1038/nmeth.2019 22743772 PMC3855844

[96] Team R. R: A language and environment for statistical computing. Vienna, Austria. 2023.

[97] Bordi C, Lamy M-C, Ventre I, Termine E, Hachani A, Fillet S, et al. Regulatory RNAs and the HptB/RetS signalling pathways fine-tune *Pseudomonas aeruginosa* pathogenesis. Mol Microbiol. 2010;76(6):1427–43. doi: 10.1111/j.1365-2958.2010.07146.x 20398205 PMC2904497

[98] Langmead B, Salzberg SL. Fast gapped-read alignment with Bowtie 2. Nat Methods. 2012;9(4):357–9. doi: 10.1038/nmeth.1923 22388286 PMC3322381

[99] Lawrence M, Huber W, Pagès H, Aboyoun P, Carlson M, Gentleman R, et al. Software for computing and annotating genomic ranges. PLoS Comput Biol. 2013;9(8):e1003118. doi: 10.1371/journal.pcbi.1003118 23950696 PMC3738458

[100] Team RC. A language and environment for statistical computing. 2021. Available from: https://www.R-project.org/

[101] Love MI, Huber W, Anders S. Moderated estimation of fold change and dispersion for RNA-seq data with DESeq2. Genome Biol. 2014;15(12):550. doi: 10.1186/s13059-014-0550-8 25516281 PMC4302049

[102] Keggrest B T. Client-side REST access to the Kyoto Encyclopedia of Genes and Genomes (KEGG) R package version 1420. 2023. doi: 10.18129/B9.bioc.KEGGREST

[103] Livak KJ, Schmittgen TD. Analysis of relative gene expression data using real-time quantitative PCR and the 2(-Delta Delta C(T)) Method. Methods. 2001;25(4):402–8. doi: 10.1006/meth.2001.1262 11846609

[104] Wickham H, Averick M, Bryan J, Chang W, McGowan L, François R, et al. Welcome to the Tidyverse. JOSS. 2019;4(43):1686. doi: 10.21105/joss.01686

[105] Kassambara A. ggpubr: ‘ggplot2’ Based Publication Ready Plots. In: 0.6.0 Rpv, editor. 2023.

[106] Crooks GE, Hon G, Chandonia J-M, Brenner SE. WebLogo: a sequence logo generator. Genome Res. 2004;14(6):1188–90. doi: 10.1101/gr.849004 15173120 PMC419797

