# Supplementary material for: Critical functions and key interactions mediated by the RNase E scaffolding domain in Pseudomonas aeruginosa
Source: PLoS Genet. 2025 Mar 17;21(3):e1011618. doi: 10.1371/journal.pgen.1011618 (PMC11964227; doi:10.1371/journal.pgen.1011618)
Supplement: S1 Table — (DOCX) [file pgen.1011618.s001.docx]

**S1 Table: List of strains used in this study**

| Strains | Genotypes / description | Source |
| --- | --- | --- |
| *E. coli* |  |  |
| Rosetta (DE3) | F- ompT hsdSB(rB- mB-) gal dcm (DE3) pRARE (Cm^R^) | Novagen |
| DH5α | recA1 endA1 hsdR17 supE44 thi-1 gyrA96 relA1 Δ(lacZYA-argF)U169 [Φ80dlacZM15]F- NaIr |  |
| HB101 | proA2 hsdS20(rB- mB-) recA13 ara-14 lacYI galK2 rpsL20 supE44 xyl-5 mtl-1 F- |  |
| BTH101 | F-, cya-99, araD139, galE15, galK16, rpsL1 (Str r), hsdR2, mcrA1, mcrB1. | Euromedex |
|  |  |  |
| *P. aeruginosa* |  |  |
| PAO1 | Wild-type |  |
| PAO1 *rne588* | Chromosonal deletion of nucleotides encoding residues 589-1057 in *rne* | This study |
| PAO1 *rne529* | Chromosonal deletion of nucleotides encoding residues 530-1057 in *rne* | This study |
| PAO1 *rne*AR1^mut^ | Chromosomal arginine-to-alanine mutations within the *rne* AR1 SLiM (see Table S4) | This study |
| PAO1 *rne*NDPR^mut^ | Chromosomal point mutation of conserved residues to GGSG within the *rne* NDPR SLiM (see Table S4) | This study |
| PAO1 *rne*AR1+AR4+REER^mut^ | Chromosomal arginine-to-alanine mutations within the *rne* AR1, AR4, and REER-repeats SLiMs (see Table S4) | This study |
| PAO1 *rne::2xStrep* | Chromosomal insertion of a Twin Strep-tag immediately upstream of the stop codon in *rne* | This study |
| PAO1 *rne529::2xStrep* | Chromosonal deletion of nucleotides encoding residues 530-1057 and insertion of a Twin-Strep tag immediately upstream of the stop codon in *rne* | This study |
| PAO1 *rne588::2xStrep* | Chromosonal deletion of nucleotides encoding residues 589-1057 and insertion of a Twin-Strep tag immediately upstream of the stop codon in *rne* | This study |
| PAO1 *rne*AR1^mut^*::2xStrep* | Chromosomal arginine-to-alanine mutations within the *rne* AR1 SLiM (see Table S4) in the strain *rne*::*2xStrep* | This study |
| PAO1 r*ne*AR1+AR4+REER^mut^*::2xStrep* | Chromosomal arginine-to-alanine mutations within the *rne* AR1, AR4, and REER-repeats SLiMs (see Table S4) in the strain *rne*::*2xStrep* | This study |
| PAO1 *rne::msfGFP* | Chromosomal insertion of *msfGFP* with a flexible linker immediately upstream of the stop codon in *rne* | This study |
| PAO1 *rne588::msfGFP* | Chromosonal deletion of nucleotides encoding residues 589-1057 and insertion of *msfgfp* with a flexible linker immediately upstream of the stop codon in *rne* | This study |
| PAO1 *rne529::msfGFP* | Chromosonal deletion of nucleotides encoding residues 530-1057 and insertion of *msfgfp* with a flexible linker immediately upstream of the stop codon in *rne* | This study |
| PAO1 *rne608::msfGFP* | Chromosomal deletion of nucleotides encoding residues 609-1057 and insertion of msfGFP with a flexible linker immediately upstream of the stop codon in *rne* | This study |
| PAO1 *rne733::msfGFP* | Chromosomal deletion of nucleotides encoding residues 734-1057 and insertion of msfGFP with a flexible linker immediately upstream of the stop codon in *rne* | This study |
| PAO1 *rne793::msfGFP* | Chromosomal deletion of nucleotides encoding residues 794-1057 and insertion of msfGFP with a flexible linker immediately upstream of the stop codon in *rne* | This study |
| PAO1 *rne940::msfGFP* | Chromosomal deletion of nucleotides encoding residues 941-1057 and insertion of msfGFP with a flexible linker immediately upstream of the stop codon in *rne* | This study |
| PAO1 *rne*AR1^mut^::*msfGFP* | Chromosomal arginine-to-alanine mutations within the *rne* AR1 SLiM (see Table S4) in the strain *rne::msfGFP* | This study |
| PAO1 *rne*AR4^mut^::*msfGFP* | Chromosomal arginine-to-alanine mutations within the *rne* AR4 SLiM (see Table S4) in the strain *rne::msfGFP* | This study |
| PAO1 *rne*AR1+AR4^mut^ ::*msfGFP* | Chromosomal arginine-to-alanine mutations within the *rne* AR1 and AR4 SLiMs (see Table S4) in the strain *rne::msfGFP* | This study |
| PAO1 *rne*REER^mut^::*msfGFP* | Chromosomal arginine-to-alanine mutations within the *rne* REER-repeats SLiM (see Table S4) in the strain *rne::msfGFP* | This study |
| PAO1 *rne*AR1+AR4+REER^mut^ ::*msfGFP* | Chromosomal arginine-to-alanine mutations within the *rne* AR1, AR4, and REER-repeats SLiMs (see Table S4) in the strain *rne::msfGFP* | This study |
| PAO1 *rne*NDPR^mut^::*msfGFP* | Chromosomal point mutation of conserved residues to GGSG within the *rne* NDPR SLiM (see Table S4) in the strain *rne::msfGFP* | This study |
| PAO1 *pnp::msfGFP*/ *rne::mCherry* | Chromosomal insertion of *msfGFP* and *mCherry* with a flexible linker immediately upstream of the stop codon in *pnp* and *rne*, respectively | This study |
| PAO1 *rhl::msfGFP*/  *rne::mCherry* | Chromosomal insertion of *msfGFP* and *mCherry* with a flexible linker immediately upstream of the stop codon in *rhl* and *rne*, respectively | This study |
| PAO1 *pnp::msfGFP* | Chromosomal insertion of *msfGFP* with a flexible linker immediately upstream of the stop codon in *pnp* | This study |
| PAO1 *rhl::msfGFP* | Chromosomal insertion of *msfGFP* with a flexible linker immediately upstream of the stop codon in *rhl* | This study |
| PAO1 *pnp::msfGFP*/ *rne529* | Chromosomal insertion of *msfGFP* with a flexible linker immediately upstream of the stop codon in *pnp* in the strain *rne529* | This study |
| PAO1 *rhl::msfGFP*/ *rne529* | Chromosomal insertion of *msfGFP* with a flexible linker immediately upstream of the stop codon in *rhl* in the strain *rne529* | This study |
| PAO1 *pnp::msfGFP*/ *rne*AR1^mut^ | Chromosomal insertion of *msfGFP* with a flexible linker immediately upstream of the stop codon in *pnp* in the strain *rne*AR1mut | This study |
| PAO1 *rhl::msfGFP*/ *rne*AR1^mut^ | Chromosomal insertion of *msfGFP* with a flexible linker immediately upstream of the stop codon in *rhl* in the strain *rne*AR1mut | This study |
| PAO1 *pnp::msfGFP*/ *rne*NDPR^mut^ | Chromosomal insertion of *msfGFP* with a flexible linker immediately upstream of the stop codon in *pnp* in the strain *rne*NDPR^mut^ | This study |
| PAO1 *rhl::msfGFP*/ *rne*NDPR^mut^ | Chromosomal insertion of *msfGFP* with a flexible linker immediately upstream of the stop codon in *rhl* in the strain *rne*NDPR^mut^ | This study |
| PAO1 *arcB::msfGFP*/*rne::mCherry* | Chromosomal insertion of msfGFP and mCherry with a flexible linker immediately upstream of the stop codon in *arcB* and *rne*, respectively | This study |

|  |
| --- |
