# Supplementary material for: Critical functions and key interactions mediated by the RNase E scaffolding domain in Pseudomonas aeruginosa
Source: PLoS Genet. 2025 Mar 17;21(3):e1011618. doi: 10.1371/journal.pgen.1011618 (PMC11964227; doi:10.1371/journal.pgen.1011618)
Supplement: S2 Table — (DOCX) [file pgen.1011618.s002.docx]

**S2 Table: List of plasmids used and cloning details.**

| Name | Used for… | Insert(s) amplified with: | Template used in PCR |
| --- | --- | --- | --- |
| pME3087_RNase E1-588 | Construction of PAO1 *rne588* | p337-p338; p339-p340 | PA01gDNA |
| pME3087_RNase E1-529 | Construction of PAO1 rne529 | p337-p341; p340-p342 | PA01gDNA |
| pME3087_RNase E1-588-msfGFP | Construction of PAO1 *rne588*::*msfGFP* | p365-p368; p369-p322; p323-p324 | pM13; PA01gDNA |
| pME3087_RNase E1-529-msfGFP | Construction of PAO1 rne529::*msfGFP* | p365-p366; p367-p322; p323-p324 | pM13; PA01gDNA |
| pEXG2_RNase EAR1mut | Construction of PAO1 *rneAR1mut*, PAO1 rne AR1mut::*msfGFP*, PAO1 rneAR1mut*:*:2xStrep | p660-p661 | pSmt3_RNase E _Cter_AR1mut-SF |
| pEXG2_RNase ENDPRmut | Construction of PAO1 *rneNDPRmut*, *rneNDPRmut*::*msfGFP* | p662-p663; p664-p665 | pSmt3_RNase E_Cter_NDPRmut-SF; PA01 gDNA |
| pEXG2_RNase EAR4mut | Construction of PAO1 *rneAR4mut*::*msfGFP* | p726-p727; p728-p729; p730-p731 | pSmt3_RNase E_Cter_AR4mut-SF; PAO1 gDNA |
| pEXG2_RNase EREERmut | Construction of PAO1 *rneREERmut*::*msfGFP* | p720-p721; p722-p723; p724-p725 | pSmt3_RNase E_Cter_REERmut-SF; PAO1 gDNA |
| pEXG2_RNase EAR1+AR4mut | Construction of PAO1 *rneAR1+AR4mut*::*msfGFP* | p726-p727; p728-p729; p730-p731 | pSmt3_RNase E _Cter_AR1+AR4mut-SF;  PAO1 gDNA |
| pEXG2_RNase EAR1+AR4+REERmut | Construction of PAO1 *rneAR1+AR4+REERmut*, PAO1 rne AR1+AR4+REERmut::*msfGFP*, PAO1 *rneAR1+AR4+REERmut*::*2xStrep* | p666-p667; p668-p669 | pSmt3_RNase E _Cter_AR1+AR4+REERmut-SF; PAO1 gDNA |
| pME3087_RNase E-2xStrep | Construction of PAO1 *rne::2xStrep* | p370-p379; p380-p381 | pSmt3-RNase E1-1057-SF; PAO1 gDNA |
| pME3087_RNase E1-529-2xStrep | Construction of PAO1 *rne529::2xStrep* strain | p378-p379; p380-p381 | pSmt3-RNase E1-529-SF; PAO1 gDNA |
| pME3087_RNase E1-588-2xStrep | Construction of PAO1 *rne588::2xStrep* strain | p378-p379; p380-p381 | pSmt3-RNase E1-588-SF; PAO1 gDNA |
| pME3087_RNase E-msfGFP | Construction of PAO1 *rne::msfGFP* strain | p319-p320; p321-p322; p323-p324 | PAO1 gDNA; msfGFP-containing plasmid |
| pME3087_RNase E-mCherry | Construction of PAO1 *rne::mCherry* strain | p319-p325; p326-p327; p328-p324 | PAO1 gDNA; mCherry-containing plasmid |
| pME3087_PNPase-msfGFP | Construction of PAO1 *pnp::msfGFP*, *pnp::msfGFP*/ *rne::mCherry,* *pnp::msfGFP* / *rne529*, *pnp::msfGFP*/*rneAR1mut*, *pnp::msfGFP*/*rneNDPRmut* strains | p405-p406; p407-p408; p409-p410 | PAO1 gDNA; msfGFP-containing plasmid |
| pME3087_RhlB-msfGFP | Construction of PAO1 *rhl::msfGFP, rhl::msfGFP/rne::mCherry,* *rhl::msfGFP/rne529*; *rhl::msfGFP/rneAR1mut*, *rhl::msfGFP/rneNDPRmut* strains | p372-p439; p441-p442; p440-p377 | PAO1 gDNA; msfGFP-containing plasmid |
| pME3087_RNase E1-608-msfGFP | PAO1 *rne608::msfGFP* strain | p365-p450; p455-p322; p323-p381 | PAO1 gDNA; msfGFP-containing plasmid |
| pME3087_RNase E1-733-msfGFP | PAO1 *rne733::msfGFP* strain | p422-p451; p456-p322; p323-p381 | PAO1 gDNA; msfGFP-containing plasmid |
| pME3087_RNase E1-793-msfGFP | PAO1 *rne793::msfGFP* strain | p422-p452; p457-p322; p323-p381 | PAO1 gDNA; msfGFP-containing plasmid |
| pME3087_RNase E1-940-msfGFP | PAO1 *rne940::msfGFP* strain | p422-p453; p458-p322; p323-p381 | PAO1 gDNA; msfGFP-containing plasmid |
| pKT25 | BTH assay |  |  |
| pKT25_PNPase | BTH assay | p240-p241 | PAO1 gDNA |
| pKT25_RhlB | BTH assay | p201-p202 | PAO1 gDNA |
| pKT25_GdhB | BTH assay | p469-p471; p470-p472 | PAO1 gDNA |
| pKT25_RpoB | BTH assay | p502-p503 | PAO1 gDNA |
| pKT25_ArcB | BTH assay | p463-p464 | PAO1 gDNA |
| pKT25_RpoC | BTH assay | p915-p916 | PAO1 gDNA |
| pKT25_RplL | BTH assay | p917-918 | PAO1 gDNA |
| pUT18C | BTH assay |  |  |
| pUT18C_RNase E1-1057 | BTH assay | p257-p166 | PAO1 gDNA |
| pUT18C_RNase E1-1025 | BTH assay | p257-p438 | PAO1 gDNA |
| pUT18C_RNase E1-940 | BTH assay | p257-p415 | PAO1 gDNA |
| pUT18C_RNase E1-793 | BTH assay | p257-p414 | PAO1 gDNA |
| pUT18C_RNase E1-733 | BTH assay | p257-p413 | PAO1 gDNA |
| pUT18C_RNase E1-608 | BTH assay | p257-p412 | PAO1 gDNA |
| pUT18C_RNase E1-588 | BTH assay | p257-p411 | PAO1 gDNA |
| pUT18C_RNase E1-529 | BTH assay | p257-p295 | PAO1 gDNA |
| pUT18C_RNase E940-1057 | BTH assay | p449-p166 | PAO1 gDNA |
| pUT18C_RNase E733-793 | BTH assay | p165-p414 | PAO1 gDNA |
| pUT18C_RNase E733-761 | BTH assay | p165-p590 | PAO1 gDNA |
| pUT18C_RNase E762-793 | BTH assay | p589-p414 | PAO1 gDNA |
| pME6032 |  |  |  |
| pME6032_RNase E | Complementation assay | p403-p790 | pME6032_RNase E-msfGFP |
| pME6032_RNase E-msfGFP | Complementation assay | p403-p404? | PAO1 *rne::msfGFP* gDNA |
| pME6032_RNase E-mCherry | Complementation assay | p403-p791 | pSmt3_RNase E-mCherry |
| pSmt3_RNase E1-529-SF | Protein purification | p181-p185 | PAO1 gDNA |
| pSmt3_RNase E1-588-SF | Protein purification | p181-p396 | PAO1 gDNA |
| pSmt3_RNase E572-1057-SF | Protein purification | p182-481 | PAO1 gDNA |
| pSmt3_RNase E572-1057^_AR4mut-SF^ | Protein purification | p555-p557; p558-p559; p560-p561; p564-p565; p562-p563; p566-p568 | PA01 gDNA; AR4mut |
| pSmt3_RNase E572-1057^_AR1mut-SF^ | Protein purification | p555-p557; p558-p559; p560-p561; p564-p565; p562-p563; p566-p568 | PA01 gDNA; AR1mut |
| pSmt3_RNase E572-1057^_REERmut-SF^ | Protein purification | p555-p557; p558-p559; p560-p561; p564-p565; p562-p563; p566-p568 | PA01 gDNA; REERmut |
| pSmt3_RNase E572-1057^_AR1+AR4mut-SF^ | Protein purification | p555-p557; p558-p559; p560-p561; p564-p565; p562-p563; p566-p568 | PA01 gDNA; AR4mut; AR1mut |
| pSmt3_RNase E572-1057^_AR1+AR4+REERmut-SF^ | Protein purification | p555-p557; p558-p559; p560-p561; p564-p565; p562-p563; p566-p568 | PA01 gDNA; AR4mut; AR1mut ; REERmut |
| pSmt3_RNase E572-1057^_NDPRmut-SF^ | Protein purification | p555-p557; p558-p559; p560-p561; p564-p565; p562-p563; p566-p568 | PA01 gDNA; NDPR |
| pSmt3-PNPase | Protein purification | p244-p245 | PAO1 gDNA |
| pSmt3-RhlB | Protein purification | p498-p499 | PAO1 gDNA |
|  |  |  |  |
| pEXG2_ArcB-  msfGFP | Construction of PAO1  *arcB::msfGFP*/*rne::mCherry* | p828-p829, p830-p831, p832-p833 | PAO1 gDNA; msfGFP-containing plasmid |
| pSmt3-ArcB | Protein purification | p834-p835 |  |
