# Supplementary material for: Critical functions and key interactions mediated by the RNase E scaffolding domain in Pseudomonas aeruginosa
Source: PLoS Genet. 2025 Mar 17;21(3):e1011618. doi: 10.1371/journal.pgen.1011618 (PMC11964227; doi:10.1371/journal.pgen.1011618)
Supplement: S3 Table — (DOCX) [file pgen.1011618.s003.docx]

**S3 Table: List of primers used in this study.**

| Primer code | Primer sequence (5' -> 3') |
| --- | --- |
| p165 | GTACGGATCCCGCCCTGGAAGCCGAGGCATTG |
| p166 | GATCGAATTCTCAGGCATGCGGGCGCG |
| p181 | GTACGGATCCAAAAGAATGCTGATCAACGCGACTC |
| p182 | CTAGGCGGCCGCGGCATGCGGGCGCGA |
| p185 | CTAGGCGGCCGCAGCGACGGTCTTGACTGC |
| p201 | GTACGGATCCCCTCAAAGCACTCAAGAAAATCTTCG |
| p202 | GATCGAATTCTCAGTGCTTGCGCGGTAC |
| p240 | GTACGGATCCCAACCCGGTAACCAAGCAATTC |
| p241 | GATCGAATTCTTACACGCCGGAAGCTTC |
| p244 | CAGTGGATCCGGAGGAAGCAACCCGGTAACCAAGCAATTC |
| p245 | CATGGAGCTCTTACACGCCGGAAGCTTCG |
| p253 | GTTTTTTTTAATACGACTCACTATAGGGACCAAGTAATGCTGTGAAATGCC |
| p254 | TGACATCCATGGGGTTCTTCC |
| p257 | GTACGGATCCCAAAAGAATGCTGATCAACGCGACTC |
| p295 | GATCGAATTCTCAAGCGACGGTCTTGACTGC |
| p319 | TAGGAGCATAGAATTCGCAAGCCACTGAAGAAGCTCC |
| p320 | CTTCACCTTTACGGGAACCACCACCGGCATGCGGGCGCGA |
| p321 | CGCCCGCATGCCGGTGGTGGTTCCCGTAAAGGTGAAGAACTG |
| p322 | CCTTTTTTCAGCCTGTCACTTATTTGTAGAGTTCATCCA |
| p323 | GATGAACTCTACAAATAAGTGACAGGCTGAAAAAAGG |
| p324 | GAATGGCAAAAGCTTCTTCAAGAACCCGCTGGTG |
| p325 | CTCCTCGCCCTTGCTCACGGAACCACCACCGGCATGCGGGCGCGA |
| p326 | CGCCCGCATGCCGGTGGTGGTTCCGTGAGCAAGGGCGAGGAG |
| p327 | CCTTTTTTCAGCCTGTCACTTACTTGTACAGCTCGTCCATG |
| p328 | CATGGACGAGCTGTACAAGTAAGTGACAGGCTGAAAAAAGG |
| p337 | TAGGAGCATAGAATTCCGACGTCGAGTCGCTGTC |
| p338 | TCAGCCTGTCACTCAGCGGGTTTGGCGCTCG |
| p339 | GCCAAACCCGCTGAGTGACAGGCTGAAAAAAGG |
| p340 | GAATGGCAAAAGCTTGCAGCTTCTTCAAGAACC |
| p341 | CAGCCTGTCACTCAAGCGACGGTCTTGACTGC |
| p342 | CAAGACCGTCGCTTGAGTGACAGGCTGAAAAAAGG |
| p365 | TAGGAGCATAGAATTCCCTGATCGTCATCGACTTCATCG |
| p366 | CAGTTCTTCACCTTTACGGGAACCACCACCAGCGACGGTCTTGACTGC |
| p367 | CAAGACCGTCGCTGGTGGTGGTTCCCGTAAAGGTGAAGAACTG |
| p368 | CAGTTCTTCACCTTTACGGGAACCACCACCGCGGGTTTGGCGCTCG |
| p369 | GCCAAACCCGCGGTGGTGGTTCCCGTAAAGGTGAAGAACTG |
| p370 | TAGGAGCATAGAATTCGAGCTGGAAGGCAGCGAG |
| p372 | TAGGAGCATAGAATTCGAGGTGCACCTGGACATGG |
| p377 | GAATGGCAAAAGCTTGCATCGCAGAGCAGCAAGG |
| p378 | TAGGAGCATAGAATTCGCAGACCAACCTGGAAGC |
| p379 | CCTTTTTTCAGCCTGTCACTCATCCGCTAGCTCCTTTCTCG |
| p380 | GGAGCTAGCGGATGAGTGACAGGCTGAAAAAAGG |
| p381 | GAATGGCAAAAGCTTGCAGCTTCTTCAAGAACC |
| p403 | GATCGAATTCATGAAAAGAATGCTGATCAAC |
| p404 | GATCGGTACCTTATTTGTAGAGTTCATCCATG |
| p405 | CAATATAGGAGCATAGAATTCGACATCAAGATCAACGGTATCAC |
| p406 | CCTTTACGGGAACCACCACCCACGCCGGAAGCTTCG |
| p407 | CGAAGCTTCCGGCGTGGGTGGTGGTTCCCGTAAAGGTGAAGAACTG |
| p408 | TTCATCGATCACGACTCGACTTATTTGTAGAGTTCATCCA |
| p409 | TGGATGAACTCTACAAATAAGTCGAGTCGTGATCGATGAAAAC |
| p410 | GAGAATGGCAAAAGCTTGGCTTTCACGCCCTTGATG |
| p411 | GATCGAATTCTCAGCGGGTTTGGCGCTCGG |
| p412 | GATCGAATTCTCAATTGCCATCGCGGCCATC |
| p413 | GATCGAATTCTCAGGCCGCGGCGTCGCGAG |
| p414 | GATCGAATTCTCACGCGGCGGCGTTATCGGTC |
| p415 | GATCGAATTCTCAGATGGCCGGGGTCGGCTC |
| p422 | TAGGAGCATAGAATTCGCAGTCAAGACCGTCGCTC |
| p438 | GATCGAATTCTCATTCCTGGGCTTCGTCCGCCTG |
| p439 | CCTTTACGGGAACCACCACCGTGCTTGCGCGGTACCGGCT |
| p440 | TGGATGAACTCTACAAATAAACGCAGGCATGGAGAA |
| p441 | AGCCGGTACCGCGCAAGCACGGTGGTGGTTCCCGTAAAGGTGAAGAACTG |
| p442 | TTTTTCTCCATGCCTGCGTTTATTTGTAGAGTTCATCCA |
| p449 | GTACGGATCCCGAGCCGACCCCGGCCATC |
| p450 | CAGTTCTTCACCTTTACGGGAACCACCACCATTGCCATCGCGGCCATC |
| p451 | CAGTTCTTCACCTTTACGGGAACCACCACCGGCCGCGGCGTCGCGAG |
| p452 | CAGTTCTTCACCTTTACGGGAACCACCACCCGCGGCGGCGTTATCGGTC |
| p453 | CAGTTCTTCACCTTTACGGGAACCACCACCGATGGCCGGGGTCGGCTC |
| p455 | GATGGCCGCGATGGCAATGGTGGTGGTTCCCGTAAAGGTGAAGAACTG |
| p456 | CTCGCGACGCCGCGGCCGGTGGTGGTTCCCGTAAAGGTGAAGAACTG |
| p457 | GACCGATAACGCCGCCGCGGGTGGTGGTTCCCGTAAAGGTGAAGAACTG |
| p458 | GAGCCGACCCCGGCCATCGGTGGTGGTTCCCGTAAAGGTGAAGAACTG |
| p463 | GTACAGATCTCGCTTTCAACATGCACAACC |
| p464 | GATCGAATTCTCAGATGTCGGCGAGG |
| p469 | CTGCAGGGTCGACTCTAGAGGCGTTCTTCACCGCG |
| p470 | GTACGAGATCTTCGTCTACTC |
| p471 | GAGTAGACGAAGATCTCGTAC |
| p472 | ACGTTGTAAAACGACGGCCGTCAGGGGATGCAGACAC |
| p481 | GTACGGATCCGCCGCCAAGCCTGCTGAAAC |
| p498 | CAGTAAGCTTCAGTGCTCAAAGCACTCAAGAAAATC |
| p499 | CATGCTCGAGTCAGTGCTTGCGCGGTAC |
| p502 | GTACGGATCCCATGGCTTACTCATACACTG |
| p503 | GATCCAATTGTTATTCGGTTTCCAGTTCGAT |
| p555 | GGATCCGCCGCCAAGCCTGCTGAAAC |
| p557 | CTCTTCGTCGCGGCGAT |
| p558 | GGCAATCGCCGCGAC |
| p559 | TGCCTCGGCTTCCAGGG |
| p560 | GACGCCGCGGCCC |
| p561 | CGCGGCGGCGTTATCGG |
| p562 | CTGCTGGTCGCGCAAG |
| p563 | GGCCGGCACCGCC |
| p564 | GGCAGCGAGGCGACC |
| p565 | AGCTTCTTCAGTGGCTTGCG |
| p566 | GAGGAGCCGACCCCGG |
| p568 | GCGGCCGCGGCATGCGGGCGCGA |
| p589 | GTACGGATCCCTCCCGCGGCCAGCGTCGT |
| p590 | GATCGAATTCTCAGCGGCGGCGCGGGCGCTC |
| p660 | GTCGACTCTAGAGGATCCGCCGCCAAGCCTGCTGA |
| p661 | GAAATTAATTAAGGTACCGAATTCCGCTTCGGCTGCTGCT |
| p662 | GTCGACTCTAGAGGATCCCCGAACGACGAGAGCCT |
| p663 | TCGACGGCGGGAGCGGCCTGAGC |
| p664 | CGCTCCCGCCGTCGAGGAGATCCCG |
| p665 | GAAATTAATTAAGGTACCGAATTCGCTCATCGACAAGGGCGG |
| p666 | GTCGACTCTAGAGGATCCGGCATCGTCTGCCCGC |
| p667 | TTGGCGGCAGGTTGATCCTTGCCTGCG |
| p668 | TCAACCTGCCGCCAAGCCTGC |
| p669 | GAAATTAATTAAGGTACCGAATTCCTCGACGGCGGGAGCG |
| p720 | GTCGACTCTAGAGGATCCGGAAGAAGCCCTGAAGGACCG |
| p721 | CGATTGCCATCGCGGCCATCGCG |
| p722 | CCGCGATGGCAATCGCCGCGAC |
| p723 | TTCGGCAATGCCTCGGCTTCCAGGG |
| p724 | CGAGGCATTGCCGAACGACGAGAGCC |
| p725 | GAAATTAATTAAGGTACCGAATTCCGTTGCCGGTTGCACG |
| p726 | GTCGACTCTAGAGGATCCGACGTCGAGTCGCTGTCG |
| p727 | GCTTGCTGGTTTCAGCAGGCTTGGCG |
| p728 | GCTGAAACCAGCAAGCCGGCTGC |
| p729 | AGCGGCGCGGCGGCGTTATCGGTCG |
| p730 | CGCCGCCGCGCCGCTGAACACC |
| p731 | GAAATTAATTAAGGTACCGAATTCCGTTGCAGGCGACGTTTTT |
| p790 | GATCGGTACCTCAGGCATGCGGGCGC |
| p791 | GATCGGTACCCTACTTGTACAGCTCGTCCATG |
| p915 | CTGCAGGGTCGACTCTAGAGAAAGACTTGCTTAATCTGTTG |
| p916 | ACGTTGTAAAACGACGGCCGTTAGTTACCGCTCGAGTTCAG |
| p917 | GTACGGATCCCGCTCTGACCAACGAAGACATC |
| p918 | GATCGAATTCTTACTTGAGCTCGACTTTGGCGCCT |
| q65 | CGTCGCGGCTACAAGTTCTCCA |
| q66 | GGCTCGCGACCCATTTCCTG |
| q107 | ATCTACAAAGGCCGCATCAC |
| q109 | CTTGAAGTATTCGCGGGAGA |
| q110 | CCCGATTCTTAAGGAACATGTGTAG |
| q111 | GTCTCCTGCTTCCTGTGTTTC |
