# Supplementary material for: Critical functions and key interactions mediated by the RNase E scaffolding domain in Pseudomonas aeruginosa
Source: PLoS Genet. 2025 Mar 17;21(3):e1011618. doi: 10.1371/journal.pgen.1011618 (PMC11964227; doi:10.1371/journal.pgen.1011618)
Supplement: S4 Table — (DOCX) [file pgen.1011618.s004.docx]

**S4 Table: Sequence of mutated SLiMs**

| RNase E SLiM | Sequence |
| --- | --- |
| AR4 | RQTRQDERRNGRQQNRRRDGRDGNRRDEE |
| AR4mut | RQTAADEAANGAAQNAAADGADGNRRDEE |
| REER | RKPREERAERQPREERAERPNREERSERRREERAERPAREERQPREGREERAERTPREERQPREGREGREERSERRREERAERPAREERQPREGREERAERPAREERQPREDRQARDAA |
| REERmut | AKPAEEAAEAQPAEEAAEAPNAEEASEAAAEEAAEAPAAEEAQPAEGAEEAAEATPAEEAQPAEGAEGAEEASEAAAEEAAEAPAAEEAQPAEGAEERAEAPAAEEAQPAEDAQAADAA |
| AR1 | GERPRRRSRGQRRRSNRRERQREVSGELEGSEATDNAA |
| AR1mut | GEAPAAASAGQAAASNAAEAQAEVSGELEGSEATDNAA |
| NDPR | GRALNDPREKRRLQREAERLAREAAAAAEAAAQAA |
| NDPRmut | GRALGGSGEKRRLQRGGSGLAREGGSGAEAAAQAA |
