# Supplementary material for: Critical functions and key interactions mediated by the RNase E scaffolding domain in Pseudomonas aeruginosa
Source: PLoS Genet. 2025 Mar 17;21(3):e1011618. doi: 10.1371/journal.pgen.1011618 (PMC11964227; doi:10.1371/journal.pgen.1011618)
Supplement: S1 Fig — Alignment of the Ec rne gene with the Pa homolog (PA2976) was done using the EBLOSUM62 matrix. (DOCX) [file pgen.1011618.s007.docx]

Aligned_sequences: 2

# 1: RNE_ECOLI

# 2: PA2976

# Matrix: EBLOSUM62

# Gap_penalty: 10.0

# Extend_penalty: 0.5

#

# Length: 1167

# Identity: 530/1167 (45.4%)

# Similarity: 651/1167 (55.8%)

# Gaps: 216/1167 (18.5%)

# Score: 2133.0

#

Domains: NTER, MTS, AR, RhlB binding site (Ec), Enolase binding (Ec), AEPV rich, PNPase binding site (Ec), REERs (Pa),NDPR (Pa)

# **truncations**

#=======================================

RNE_ECOLI 1 MKRMLINATQQEELRVALVDGQRLYDLDIESPGHEQKKANIYKGKITRIE 50

||||||||||.|||||||||||||:||||||...||||||||||:|||:|

PA2976 1 MKRMLINATQPEELRVALVDGQRLFDLDIESGAREQKKANIYKGRITRVE 50

RNE_ECOLI 51 PSLEAAFVDYGAERHGFLPLKEIAREYFPANYSAHGRPNIKDVLREGQEV 100

|||||||||:|||||||||||||:|||| ..|..||.|||:||.|||||

PA2976 51 PSLEAAFVDFGAERHGFLPLKEISREYF--KKSPEGRINIKEVLSEGQEV 98

RNE_ECOLI 101 IVQIDKEERGNKGAALTTFISLAGSYLVLMPNNPRAGGISRRIEGDDRTE 150

|||::|||||||||||||||||||.||||||||||||||||||||::|.|

PA2976 99 IVQVEKEERGNKGAALTTFISLAGRYLVLMPNNPRAGGISRRIEGEERNE 148

RNE_ECOLI 151 LKEALASLELPEGMGLIVRTAGVGKSAEALQWDLSFRLKHWEAIKKAAES 200

|:|||..|..|..|||||||||:|:|.|.|||||.:.|:.|.|||:|:..

PA2976 149 LREALNGLNAPADMGLIVRTAGLGRSTEELQWDLDYLLQLWSAIKEASGE 198

RNE_ECOLI 201 RPAPFLIHQESNVIVRAFRDYLRQDIGEILIDNPKVLELARQHIAALGRP 250

|.|||||:||||||:||.||||||||||:|||:....|.|...|..: .|

PA2976 199 RGAPFLIYQESNVIIRAIRDYLRQDIGEVLIDSIDAQEEALNFIRQV-MP 247

RNE_ECOLI 251 DFSSKIKLYTGEIPLFSHYQIESQIESAFQREVRLPSGGSIVIDSTEALT 300

.::||:|||...:|||:.:|||||||:||||||:||||||||||.||||.

PA2976 248 QYASKVKLYQDSVPLFNRFQIESQIETAFQREVKLPSGGSIVIDPTEALV 297

RNE_ECOLI 301 AIDINSARATRGGDIEETAFNTNLEAADEIARQLRLRDLGGLIVIDFIDM 350

:|||||||||:||||||||..||||||:||||||||||:|||||||||||

PA2976 298 SIDINSARATKGGDIEETALQTNLEAAEEIARQLRLRDIGGLIVIDFIDM 347

RNE_ECOLI 351 TPVRHQRAVENRLREAVRQDRARIQISHISRFGLLEMSRQRLSPSLGESS 400

||.::|||||.|:|||:..||||:|:..||||||||||||||.|||||:|

PA2976 348 TPAKNQRAVEERVREALEADRARVQVGRISRFGLLEMSRQRLRPSLGETS 397

RNE_ECOLI 401 HHVCPRCSGTGTVRDNESLSLSILRLIEEEALKENTQEVHAIVPVPIASY 450

..|||||:|.|.:||.|||||:|||||||||||:.|.||.|.||..:|::

PA2976 398 GIVCPRCNGQGIIRDVESLSLAILRLIEEEALKDRTAEVRARVPFQVAAF 447

RNE_ECOLI 451 LLNEKRSAVNAIETRQDGVRCVIVPNDQMETPHYHVLRVRKGEETPTL-- 498

||||||:|:..||.| ...|..|:|:|.:||||:.|.|:| :::|.|

PA2976 448 LLNEKRNAITKIELR-TRARIFILPDDHLETPHFEVQRLR--DDSPELVA 494

RNE_ECOLI 499 ---SYMLPKL-HEEAMALPSEEEFAERKRPEQPALATFAMPDVPPAPTPA 544

||.:..: ||||..:.|...... ::.|:.|.| |.

PA2976 495 GQTSYEMATVEHEEAQPVSSTRTLVR----QEAAVKTV**A**---------PQ 531

RNE_ECOLI 545 EPAAPVVAPAPKAAPATPAAP-AQPGLLSRFFGALKALFSGGEE--TKPT 591

:| ||....||..||.| .:|.|......:|..||:|.:: .||.

PA2976 532 QP-----APQHTEAPVEPAKPMPEPSLFQGLVKSLVGLFAGKDQPAAKPA 576

RNE_ECOLI 592 EQPAPKAEAKPERQQDRRKPRQNNRR-------DRNERRDTRSERTEGS- 633

|...|.|| :..||.:||..||.||| .|:|.|..|.||.|..

PA2976 577 ETSKPAAE-RQTRQDERRNGRQQNRRRDGRDGN**R**RDEERKPREERAERQP 625

RNE_ECOLI 634 -------DNREENRRNRRQAQQQTAETRESRQQAEVTEK--ARTADEQQA 674

.|||| |..||:.::.....||.||..|..|: .||..|::.

PA2976 626 REERAERPNREE-RSERRREERAERPAREERQPREGREERAERTPREERQ 674

RNE_ECOLI 675 PRRERSRRRNDDKRQAQQEAKALNVEEQSVQETEQEERVRPVQPRRKQRQ 724

||..|..|....:|:.::.|:....||:..:|..:|...||.:..|:.|

PA2976 675 PREGREGREERSERRREERAERPAREERQPREGREERAERPAREERQPR- 723

RNE_ECOLI 725 LNQKVRYEQSVAEEAVVAPVVEETVAAEPIVQEAPA-PRTELVKVPLPVV 773

|:..|.:....||.| |..|.:

PA2976 724 ---------------------EDRQARDAA**A**LEAEALPNDESL------- 745

RNE_ECOLI 774 AQTAPEQQEENNADNRDNGGMPRRSRRSPRHLRVSGQRRRRYRDERY--- 820

||.|:::.| |..|||..| |||||..|.||.

PA2976 746 -----EQDEQDDTD----GERPRRRSR--------GQRRRSNRRERQREV 778

RNE_ECOLI 821 ---------PTQSPMPL-TVACASPELASGKVWIRYPIVRPQDVQVEEQR 860

...:..|| |||.|: |:| :.|..:.

PA2976 779 SGELEGSEATDNAA**A**PLNTVAAAA---AAG-------------IAVASEA 812

RNE_ECOLI 861 EQEEVHVQPMVTEVPVA---AAIEPVVSAPVVEEVAGV-----------V 896

.:..|...|..|....: |:.|...|.....|..|. :

PA2976 813 VEANVEQAPATTSEAASETTASDETDASTSEAVETQGADSEANTGETADI 862

RNE_ECOLI 897 EAPVQVAEPQPEVVETTHPEVIAAAVTEQPQVITESDVAVAQEVAEQAEP 946

||||.|:..:.|..::| ::.|..||:....:|| .|..|.||.

PA2976 863 EAPVTVSVVRDEADQST---LLVAQATEEAPFASES-----VESREDAES 904

RNE_ECOLI 947 VVEPQEETADIEEVVETAEVVVAEPE-----------VVAQPA------- 978

.|:|..|.| |||.....|.||.|. :.|.||

PA2976 905 AVQPATEAA--EEVAAPVPVEVAAPSEPAATEEPTPA**I**AAVPANATGRAL 952

RNE_ECOLI 979 -------------------APVVAEVAAE----VETVAAVEPEVTVEHNH 1005

|...||.||: ||.:.||..|.......

PA2976 953 NDPREKRRLQREAERLAREAAAA**A**EAAAQAAPAVEEIPAVASEEASAQEE 1002

RNE_ECOLI 1006 ATAP----MTRAPAPEYVPEA-------PRHSDWQRPTFAFEGKGAAGGH 1044

..|| :|:|..|....|| |..| |:|||...

PA2976 1003 PAAPQAEEITQADVPSQADEAQEAVQAEPEAS----------GEGAADTE 1042

RNE_ECOLI 1045 TATHHASAAPARPQPVE 1061

.|.....:..:||..

PA2976 1043 HAKKTEESETSRPHA-- 1057
